# Physiological changes and transcriptome profiling in *Saccharum spontaneum* L. leaf under water stress and re-watering conditions

**DOI:** 10.1038/s41598-021-85072-1

**Published:** 2021-03-09

**Authors:** Changning Li, Zhen Wang, Qian Nong, Li Lin, Jinlan Xie, Zhanghong Mo, Xing Huang, Xiupeng Song, Mukesh Kumar Malviya, Manoj Kumar Solanki, Yangrui Li

**Affiliations:** 1 Key Laboratory of Sugarcane Biotechnology and Genetic Improvement (Guangxi), Ministry of Agriculture/Guangxi Key Laboratory of Sugarcane Genetic Improvement, Nanning, 530007 China; 2 College of Biology and Pharmacy, Yulin Normal University, Yulin, 537000 China; 3 Plant Protection Research Institute, Guangxi Academy of Agricultural Sciences, Nanning, 530007 China; 4 Department of Food Quality and Safety, The Volcani Center, Institute for Post-Harvest and Food Sciences, Agricultural Research Organization, Rishon LeZion, Israel

**Keywords:** Drought, Plant physiology

## Abstract

As the polyploidy progenitor of modern sugarcane, *Saccharum spontaneum* is considered to be a valuable resistance source to various biotic and abiotic stresses. However, little has been reported on the mechanism of drought tolerance in *S. spontaneum*. Herein, the physiological changes of *S. spontaneum* GXS87-16 at three water-deficit levels (mild, moderate, and severe) and after re-watering during the elongation stage were investigated. RNA sequencing was utilized for global transcriptome profiling of GXS87-16 under severe drought and re-watered conditions. There were significant alterations in the physiological parameters of GXS87-16 in response to drought stress and then recovered differently after re-watering. A total of 1569 differentially expressed genes (DEGs) associated with water stress and re-watering were identified. Notably, the majority of the DEGs were induced by stress. GO functional annotations and KEGG pathway analysis assigned the DEGs to 47 GO categories and 93 pathway categories. The pathway categories were involved in various processes, such as RNA transport, mRNA surveillance, plant hormone signal transduction, and plant-pathogen interaction. The reliability of the RNA-seq results was confirmed by qRT-PCR. This study shed light on the regulatory processes of drought tolerance in *S. spontaneum* and identifies useful genes for genetic improvement of drought tolerance in sugarcane.

## Introduction

Sugarcane (*Saccharum* spp. hybrids) is grown globally in tropical and subtropical countries for sugar production. There are currently inadequate arable lands for the growth and development of crop plants because of increasing populations and unfavorable environmental conditions. This phenomenon has increased the acreage (~ 80%) of sugarcane, especially in upland areas across the world where adequate irrigation is limited^1^. Water deficit is a critical constraint for sugarcane production. Thus, it is necessary to develop new sugarcane varieties with high water use efficiency or more tolerant of dehydration.

Plants develop numerous adaptive systems, including morphological and physiological modifications as the first defense against water deficit stress^2^. Plants may also increase the synthesis of compatible solutes such as glycine betaine, proline, trehalose, sucrose, or glucose during stress conditions^3,4^. These solutes play diverse functions such as osmotic adjustment, acting as carbon or nitrogen sources, and stabilizing proteins and membranes. Water stress induces the overproduction of reactive oxygen species (ROS), which causes a redox imbalance in the cells. This imbalance disrupts the electron transport chain, lipid peroxidation, protein denaturation, and DNA mutation^5^. Various enzymatic antioxidants, such as superoxide dismutase (SOD), peroxidase (POX), catalase (CAT), and glutathione reductase (GR), alleviate this oxidative damage^6^. However, the drought intensity and duration and plant species or cultivar differences determine the antioxidant enzyme activities under water stress. Besides ROS, plant responses to abiotic stress are mediated by phytohormones, which coordinate complex stress-adaptive signaling cascades. Notably, abscisic acid (ABA) regulates plant cellular adaptation to drought and other stresses^6,7^. Other molecular responses, such as expression of multiple genes, signal transduction, protein synthesis, compounds synthesis, and regulatory loci, also play essential roles in drought stress^8^.

Breeders have attempted to develop more stress-resistant cultivars for decades by exploring plant physiological and biochemical processes of drought tolerance. However, sugarcane improvements by conventional breeding approaches are challenged by the polyploid genetic structure. Modern sugarcane cultivars are derived from interspecific hybridization between *Saccharum officinarum* and *Saccharum spontaneum.* High sugar content traits are derived from cultivated ‘noble’ forms of *S. officinarum*, while disease resistance, stress tolerance, and ratooning capacity traits are obtained from *S. spontaneum. S. spontaneum* is backcrossed to *S. officinarum* to recover high biomass and high sugar content^9^. These cultivars have up to 130 chromosomes distributed in ~ 12 homologous or homoeologous groups, with a total genome size of ~ 10 Gb^10–12^. The large genome hampers genome sequencing, assembly, and annotation. Drought tolerance is a complex quantitative trait that involves multiple pathways, regulatory networks, cellular compartments, and multiple genes^8^. Conventional breeding strategies for crop improvement are limited by the complexity of stress-tolerance traits, lack of efficient selection techniques, and low genetic variance and fertility.

Transcriptome profiling by de novo RNA sequencing (RNA-seq) is an alternative way to identify genes and pathways related to stress responses. RNA-seq facilitates the transcriptome study of non-model plant species that lack a reference genome. It allows the discovery of virtually all expressed genes in plant tissues under abiotic stress by manipulating the characteristic of interest. It has been used to reveal pathways associated with graft healing by asymmetric profiling in tomato^13^ and alternative splicing under salt stress in cotton^14^. It has also been employed to evaluate the effect of water stress on *Arundo donax* L.^15^ and *Cicer arietinum* L.^16^ and identify the novel genes controlling aluminium tolerance in *Arabidopsis thaliana*^17,18^. It has been used to study smut infection response^19,20^, vital genes involved in cellulose and lignin biosynthesis^21^, plant growth-promoting rhizobacteria inoculation response^22^, and water, salt, and cold stress response^23–25^ in cultivated sugarcane. Transcriptome sequencing has also been extensively applied to study gene expression changes in *S. spontaneum* in response to drought stress^26,27^, cold damage^28–31^, and identification of transcription factors^32^.

Nonetheless, understanding stress perception or signaling remains relatively low despite a comprehensive knowledge of the mechanisms governing cellular response to water scarcity. Herein, the global transcriptional changes in leaves of *S. spontaneum* GXS87-16 at the elongation stage under water stress were characterized by RNA-Seq. The study aimed to identify differentially expressed genes and their associated pathways for future plant breeding programs in cultivated sugarcane and other essential grass species.

## Results

### Changes in physiological parameters under water stress and re-watering condition

**Figure 1:**
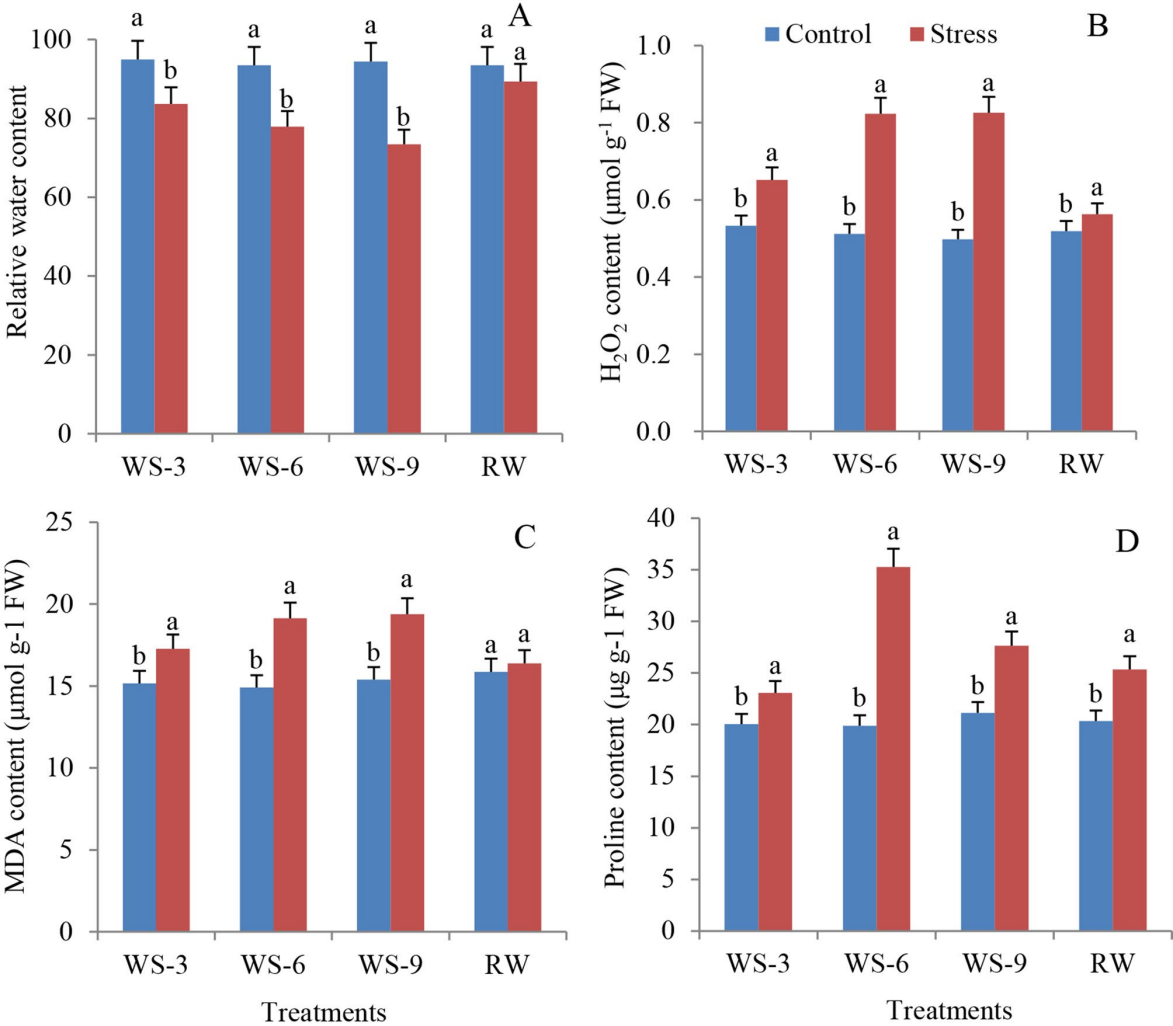
Effects of water stress and re-watering on RWC (**A**), H_2_O_2_ (**B**), MDA (**C**) and proline (**D**) content in leaves of *S. spontaneum* GXS87-16. Samples were collected at WS-3, WS-6, WS-9, and RW conditions, which corresponded to stop watering 3rd, 6th, 9th days and 4th days after re-watering, respectively. Bars with different superscript letters indicate significant differences at the *P* < 0.05 probability at the same time point.

Changes in physiological parameters were determined to verify the effects of water stress, and re-watering (RW) on *S. spontaneum* leaves. The leaf relative water content (RWC) decreased by 11.8%, 16.7%, and 22.2% at WS-3, WS-6, and WS-9 stages, respectively, compared to the control (Fig. 1A). However, the RWC was restored after RW (only 4.3% lower than control). H_2_O_2_ is a metabolic by-product of reactive oxygen that is an indicator of ROS removal ability. The H_2_O_2_ content increased by 22.3%, 60.7%, and 65.9% at WS-3, WS-6, and WS-9 conditions, respectively. However, it only increased by 8.5% after RW (Fig. 1B). The amount of MDA reflects the degree of lipid peroxidation and indirectly shows the degree of cell damage. The MDA content significantly increased under water stress compared to the control. However, it sharply decreased under RW conditions (Fig. 1C). The proline (PRO) contents under water stress were remarkably higher than those of the control. Notably, the PRO content was 77.3% higher than that of the control at WS-6. It remained significantly higher than that of control even after RW (Fig. 1D).

### Changes in plant hormone production under water stress and re-watering condition

**Figure 2:**
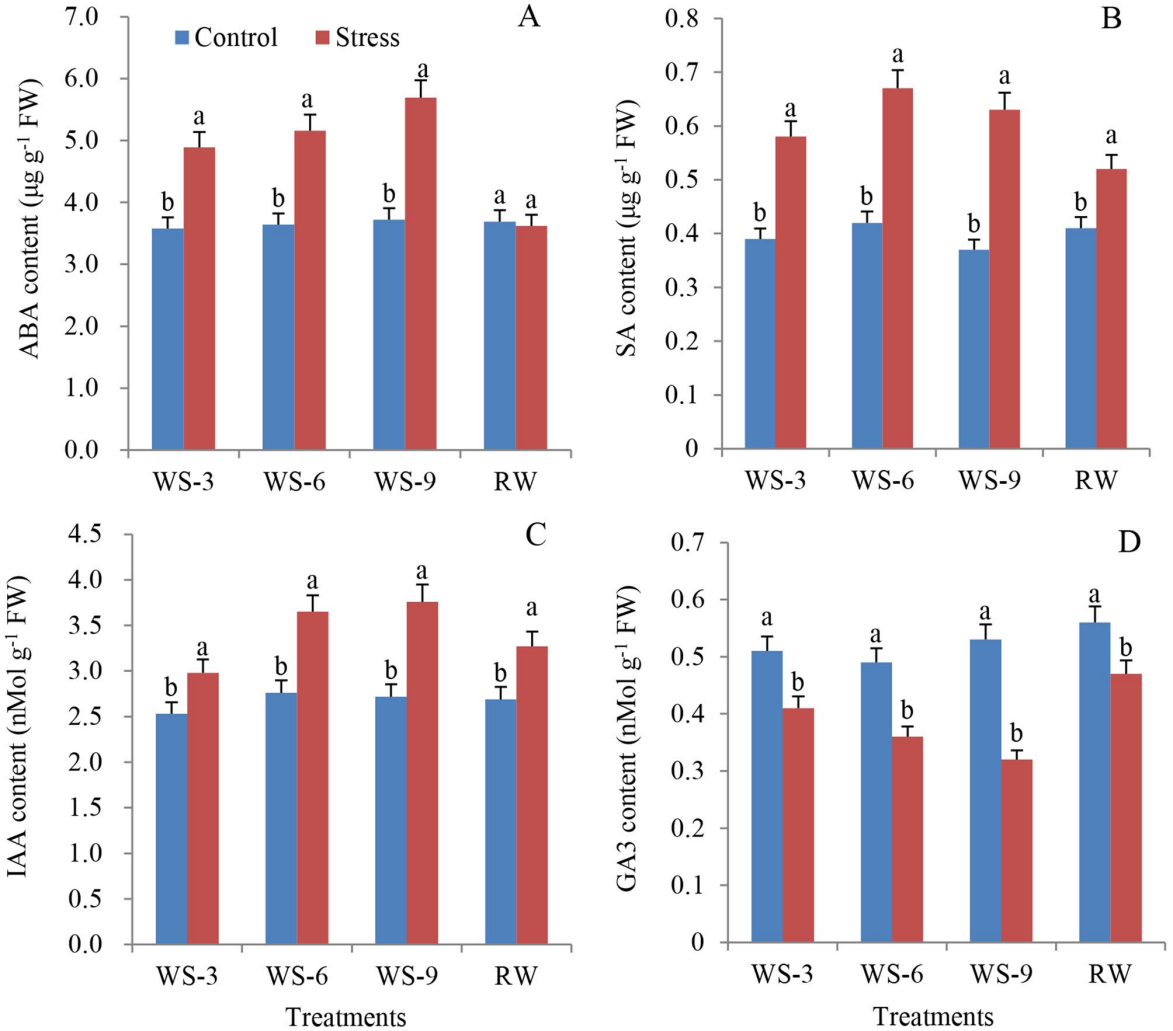
Effects of water stress and re-watering on ABA (**A**), SA (**B**), IAA (**C**) and GA3 (**D**) content in leaves of *S. spontaneum* GXS87-16. Samples were collected at WS-3, WS-6, WS-9, and RW conditions, which corresponded to stop watering 3rd, 6th, 9th days and 4th days after re-watering, respectively. Bars with different superscript letters indicate significant differences at the *P* < 0.05 probability at the same time point.

Figure 2 illustrates the effects of water stress on endogenous hormones in *S. spontaneum.* The ABA, SA, and IAA contents increased by of 36.6%, 48.7%, and 17.8% at WS-3, 41.8%, 59.5%, and 32.2% at WS-6, and 52.9%, 70.3% and 38.2% at WS-9 compared to the control (Fig. 2A–C). However, their contents were restored almost to normal after RW. In contrast, the GA3 content reduced by 19.6%, 26.5%, and 39.6% at WS-3, WS-6, and WS-9, respectively, compared to the control. Though it was restored after re-watering, it remained significantly lower than that of the control (Fig. 2D).

### Changes in activities of antioxidant enzymes under water stress and re-watering condition

**Figure 3:**
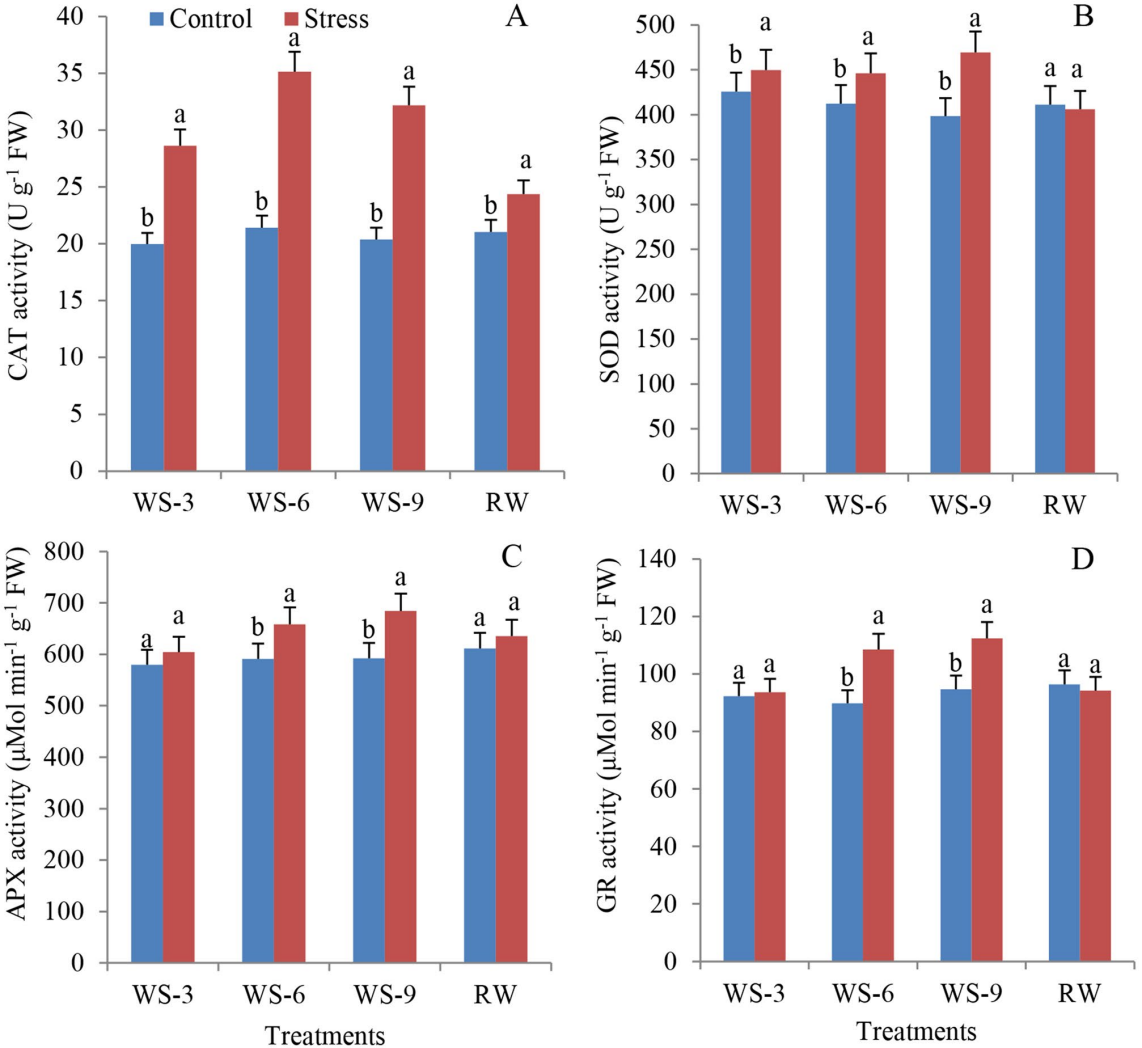
Effects of water stress and re-watering on CAT (**A**), SOD (**B**), APX (**C**) and GR (**D**) activities in leaves of *S. spontaneum* GXS87-16. Samples were collected at WS-3, WS-6, WS-9, and RW conditions, which corresponded to stop watering 3rd, 6th, 9th days and 4th days after re-watering, respectively. Bars with different superscript letters indicate significant differences at the P < 0.05 probability at the same time point.

Drought stress causes the accumulation of osmotic substances. This accumulation indicated that osmotic substances played an important role in plants’ resistance to stress. The main functions of CAT, SOD, APX, and GR in plants are to remove active oxygen under adversity and prevent membrane lipid peroxidation. The activities of CAT, SOD, APX, and GR enzymes initially increased and then decreased (Fig. 3). At WS-3, the activity of CAT and SOD was significantly activated. CAT activity reached its maximum at WS-6 with a 63.9% increase compared to the control and decreased (Fig. 3A). Similar to the PRO content, CAT activity dropped sharply after RW but remained significantly higher than that of the control. At WS-9, the activities of SOD, APX, and GR reached the maximum with a 17.8%, 15.5%, and 23.9% increase, respectively, compared to the control (Fig. 3B–D). There were no significant differences in SOD, APX, and GR activities even after RW (Fig. 3B–D).

### RNA-seq results summary

de novo RNA sequencing was used for transcriptome analysis of *S. spontaneum* GXS87-16. The number of raw and clean reads of the nine RNA-seq libraries was 83,096,344 and 79,502,382, respectively. The total clean nucleotides (Q20 > 98.66%) were 7,155,214,380 with an average GC content of 51.91%. Assembly results revealed that there were 111,978 unigenes with a full length of 98,025,473. The average length of the unigenes was 875 with an N50 of 1651. Function annotation analysis revealed that 64205, 73838, 39201, 38163, 24445, 47282 unigenes were annotated to the NR, NT, Swiss-Prot, KEGG, COG, and GO database, respectively. Among them, 78657 unigenes matched to at least one database. Protein coding prediction analysis further revealed that 61988 CDS mapped to the protein database and 4217 predicted CDS.

### Identification of differentially expressed genes

**Figure 4:**
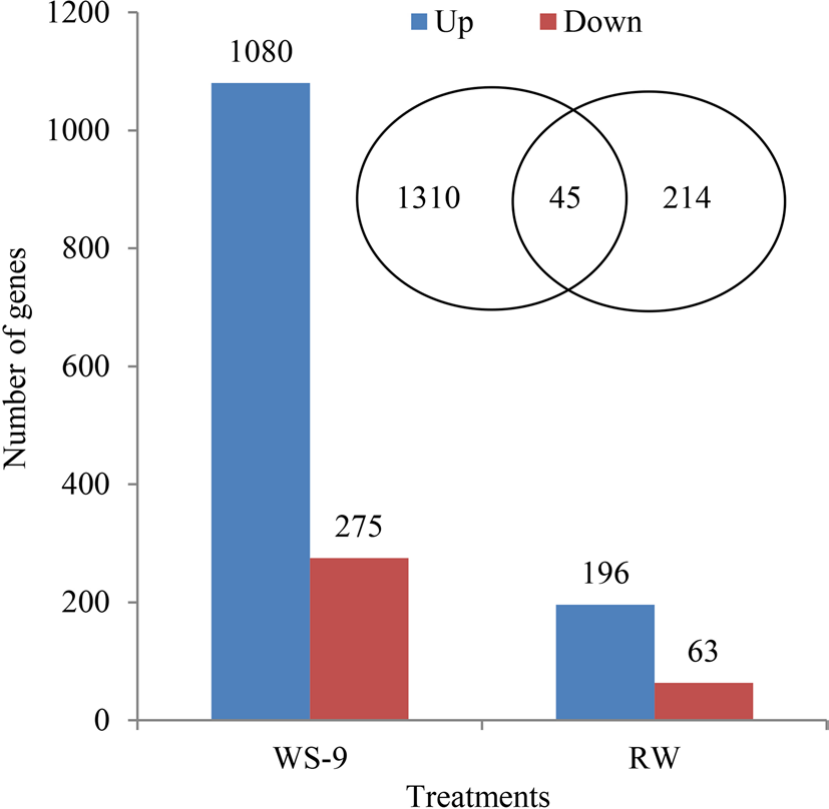
Differential gene expression profiles. Up and down indicate up and down regulated genes. Venn diagram presents the number of overlapping genes between the analyzed time points. Samples were collected at WS-9 and RW conditions, which corresponded to stop watering 9th days and 4th days after re-watering, respectively.

The relative expression levels of genes were evaluated as the fragment per kilobase of transcript sequence per million base pairs sequenced (FPKM) values. These values were calculated based on the uniquely mapped reads for the control, water-stressed, and re-watered plants. There were 1569 differentially expressed genes in response to water stress and re-watering based on comparative analysis at a *p*-value < 0.05 and |log2 fold change [L2fc]|> 2 (Fig. 4). Among them, 1310 and 214 genes were significantly expressed under drought and re-watered conditions, respectively. There were 45 genes significantly expressed overlapped in both conditions (Supplementary Fig. S1). Additionally, the DEGs were preferentially induced under stress conditions. Among the 1310 genes significantly expressed under water stress, 1080 (79.7%) genes were up-regulated, and 275 (20.3%) were downregulated (Fig. 4). Similarly, 196 (75.7%) genes were up-regulated, and 63 (24.3%) downregulated among the 214 genes significantly expressed under re-watered conditions. Notably, the number of differentially expressed genes decreased significantly after re-watering.

### GO classification of differentially expressed genes

**Figure 5:**
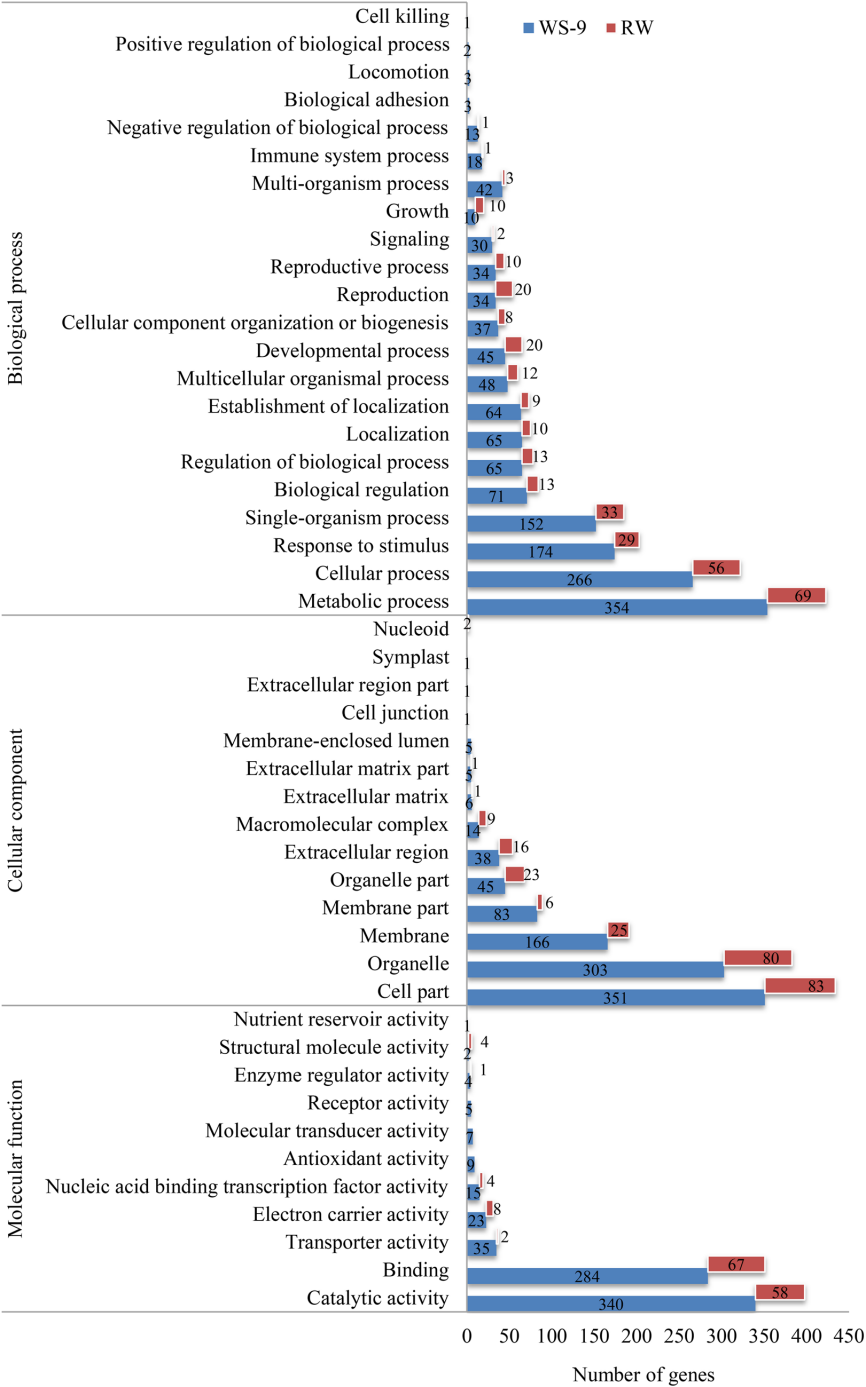
GO classification of differentially expressed genes under water stress and re-watering conditions. This subset of GO terms was processed by WEGO to categorize the genes into functional groups as follows: biological process, cellular component, and molecular function. The number of annotated stress responsive genes is shown for each treatment. Samples were collected at WS-9 and RW conditions, which corresponded to stop watering 9th days and 4th days after re-watering, respectively.

GO classification of DEGs was performed on the Web Gene Ontology Annotation Map. The DEGs were assigned to 47 GO categories (Fig. 5). Among them, 22 categories belonged to biological processes, 14 to cellular components, and 11 to molecular function. Metabolic processes, cellular processes, and stimulus–response were the top three terms amongst the DEGs in the biological process category. The cell part, organelle, and membrane categories were the top three terms amongst the DEGs in the cell components category. Catalytic activity, binding, transport, electron carrier, nucleic acid binding transcription factor, antioxidant, molecular transducer, receptor, enzyme regulator, structural molecule, and nutrient reservoir activities were the main activities of the DEGs.

### Pathway classification of differentially expressed genes

**Figure 6:**
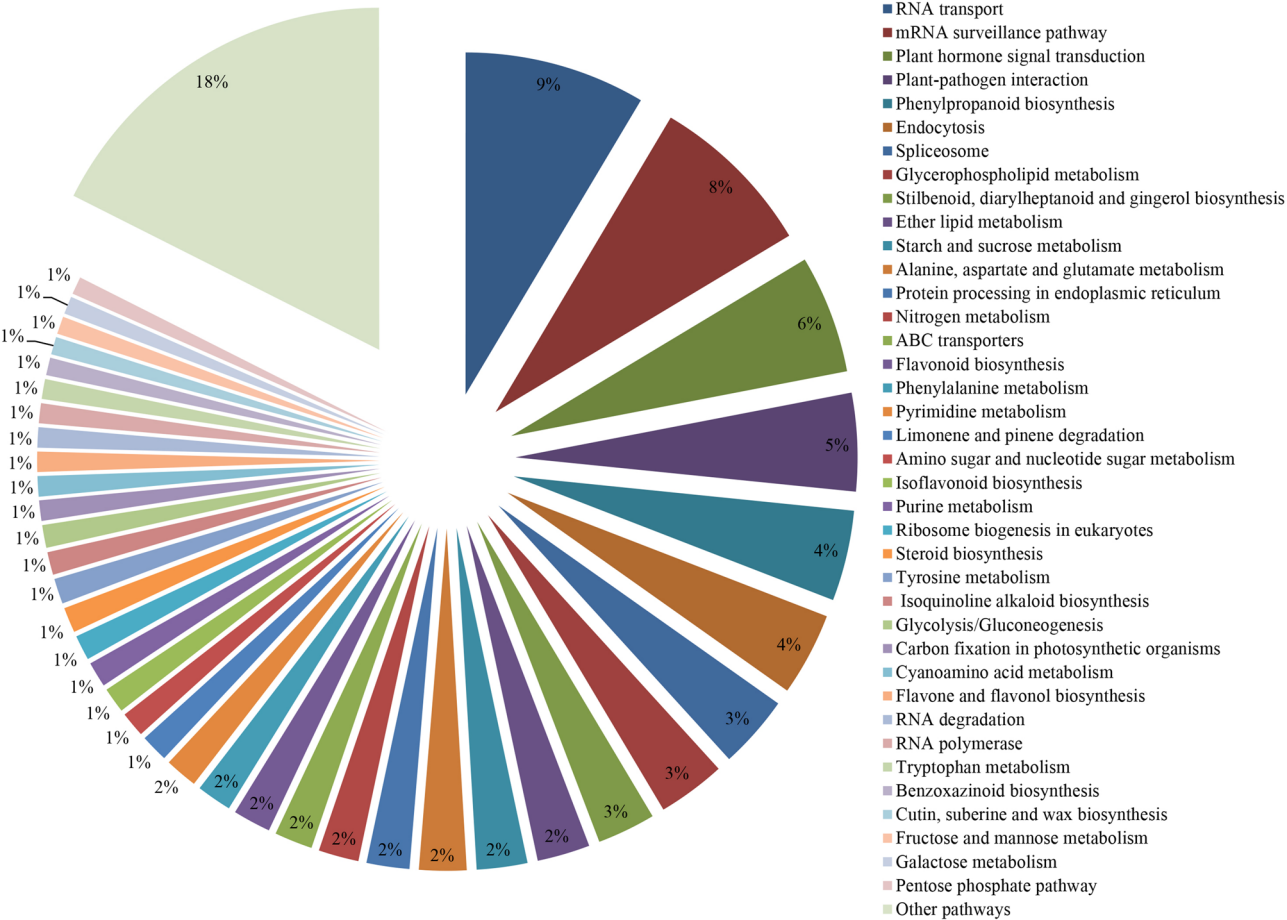
Functional classification of pathway terms for differentially expressed genes under water stress and re-watering conditions. The pathway annotations were acquired with KAAS using an e-value threshold of <10^-5^. A total of 402 differentially expressed genes were classified into 93 pathway categories. The data for pathway categories that represented less than 1% of the differentially expressed genes were included in other pathway categories.

There were 402 non-overlapping DEGs classified into 93 pathway categories by KAAS. The RNA transport pathway (9%) and the mRNA surveillance pathway (8%) were the most highly represented pathway categories. They were followed by the plant hormone signal transduction pathway (6%), which indicated that plant hormones played an important role in coping with water shortage. Other pathway categories included: plant-pathogen interaction (5%), phenylpropane biosynthesis (4%), endocytosis (4%), spliceosome (3%), glycerophospholipid metabolism (3%), stilbenoid, diarylheptanoid, and gingerol biosynthesis (3%) (Fig. 6). Genes in each pathway category are outlined in Supplementary Table S1.

### Validation of RNA-seq results by quantitative real-time PCR

To validate the RNA-seq results, fifteen DEGs were randomly selected and analyzed by real-time PCR (qRT-PCR). The expression levels of selected genes in the qRT-PCR analysis are summarized in Supplementary Table S2. Transcriptional expression levels of selected DEGs at WS-9 and RW condition in qRT-PCR were found to correlate with that of RNA-seq, indicating the accuracy transcriptome data in reflecting the in vivo transcript expression in the present study.

## Discussion

Water stress is an important factor limiting plant growth^33^. It affects various physiological and biochemical functions such as photosynthesis, chlorophyll synthesis, and nutrient metabolism, including ion absorption and transport, respiration, and carbohydrate metabolism^34,35^. Plants rapidly accumulate small molecule osmotic adjustment substances under drought stress, such as soluble sugars, proline, and betaine. Reduction in cells’ water potential improves the ability of cells to absorb water and retain water. It also maintains the swelling pressure of cells and ensures the normal functioning of plant physiological metabolisms^36^. Plants have a physiological response mechanism to respond to changes in soil moisture and tolerance to drought stress^37^. The stress is relieved after re-watering, and the plant undergoes adaptive changes in structure and physiology, quickly making up for the damage caused by the drought stress. Thus, drought positively affects plants after re-watering through a compensation effect^38,39^. Jin et al.^40^ reported that the proline, soluble sugars, and protective enzymes of *Portulaca oleracea* increased under drought stress. However, these indexes decreased after rehydration, and the plants resumed normal growth.

Accumulation of ROS leads to increased peroxidation of membrane lipids, oxidative damage to membrane components and structures, and generation of large amounts of MDA, which consequently destroys the membrane systems^41^. Herein, the longer the drought stress, the more MDA was produced. However, it was restored to normal levels after re-watering. Osmotic adjustment is an important physiological adaptation method of plants to drought^42^. Herein, the proline in the leaves accumulated at WS-6. An accumulation of H_2_O_2_ and MDA accompanied it to improve drought resistance by adjusting the osmotic potential. Intensification of drought stress caused H_2_O_2_ and MDA in the leaves to increase significantly in the first nine days. This increase indicated that drought stress caused the accumulation of ROS, resulting in membrane lipid peroxidation. Peroxidation further caused damage to the membrane, thus causing the MDA concentration and leaf damage to increase. Accumulation of MDA increases cell membrane permeability, and subsequently, extensive ion extravasation as a self-protection mechanism. Under drought stress, the concentration of proline and MDA of alfalfa (*Medicago sativa*) increased significantly, thereby reducing plant growth^43^. Herein, the plants overcame drought stress after re-watering; the water potential increased, and the regulation substances in the leaves were gradually restored to normal levels. The osmotic adjustment capacity also steadily decreased, and the content of H_2_O_2_, MDA, and proline decreased, causing the plants to resume normal growth. High levels of MDA accumulation under water stress and return to normal state during re-watering as an indication of drought tolerance in maize was reported by Chen et al.^44^.

Excessive ROS accumulation can cause oxidative damage at the cellular level, destroy cell membranes, and lead to enzyme inactivation, protein degradation, and ion imbalance in cells^45^. Nonetheless, plants can eliminate the excessive ROS produced in cells through complex non-enzymatic and enzymatic antioxidant systems^46^. Drought stress causes a sharp rise in the ROS levels of plant cells, which breaks the redox balance in plants. In the first nine days of drought stress, SOD, APX, GR, and CAT activities in *S. spontaneum* leaves gradually increased. This increase indicates that the four enzymes played a synergistic role to remove intracellular ROS. A similar phenomenon occurs when wheat is under drought stress; SOD, APX, GR, and CAT systems are up-regulated, causing the plants to adapt to drought^47^. Previous studies on sugarcane postulate that accumulation of antioxidant enzymes is a measure of its capability to self-protect against oxidative stress^33,48^. At WS-9, SOD and APX were still relatively high and continued to produce excessive ROS in the cells as the major drought resisting enzyme. However, all indicators were restored after re-watering, demonstrating that *S. spontaneum* has a strong drought tolerance ability. The antioxidant enzyme system plays an active role under drought and re-watering conditions, enabling *S. spontaneum* to maintain better cell membrane stability.

Plant endogenous hormones have a regulatory role in growth and development and response to adversity through complex mechanisms^49^. Previous studies postulate that drought stress increases the ABA content of plants and increases or decreases the IAA content^50^. For instance, both ABA and MDA levels returned to normal when *Eucalyptus* was rehydrated after three weeks of drought stress^51^. Contrary to these findings, Zhang et al. reported no significant differences in ABA content between well-watered and un-watered maize leaves^52^. Herein, drought stress and re-watering affected the endogenous hormones of *S. spontaneum*. Under drought stress, the content of ABA and IAA in the leaves increased. However, their contents decreased after re-watering to produce a compensation effect. Nonetheless, the IAA content was still significantly higher than that of the control. These findings proved that rehydration could restore ABA and IAA content caused by drought and produce different compensation effects on various endogenous hormones. SA induces the production of large amounts of ABA and proline in barley leaves^53^. In *Arabidopsis thaliana*, SA induces ROS production in the photosynthetic tissues, thereby enhancing the plant's resistance to stress^54^. Previous studies postulate that reducing GA_3_ levels can improve the drought tolerance of plants^55^. In this study, the leaf GA_3_ content was significantly lower than that of control, while the content of SA was substantially higher than that of control under drought stress. These changes increased the SOD activity of *S. spontaneum* leaves, thereby reducing cell membrane damage and enhancing the drought tolerance ability of *S. spontaneum*. An increase in SA concentration can trigger a series of physiological reactions to improve the drought resistance of wheat. These reactions include increasing the ABA and CAT contents, which lead to proline accumulation^56,57^. Similar results were obtained in this study.

Transcriptome sequencing technology is widely used to analyze plant metabolic pathways, discover new transcripts, improve genome annotation, and screen for specific functional genes under abiotic stress^58,59^. In this study, there were important changes in *S. spontaneum* transcript levels under drought stress. Pathway annotation revealed that the DEGs were mainly enriched in plant hormone signal transduction, alanine, aspartate and glutamate metabolism, protein processing in the endoplasmic reticulum, nitrogen metabolism, ABC transporters, plant-pathogen interaction, and pyrimidine metabolism. This result verified the transcriptome analysis results of sugarcane under progressive osmotic stress by Santana et al.^60^. The *cycloartenol synthase* gene is an important regulatory gene for synthesizing phytosterols, which are essential cell membrane components related to the chloroplast function^61^. Four *cycloartenol synthase* genes (Unigene28448, Unigene1402, CL13693.Contig36, Unigene28853) were up-regulated under drought conditions. This finding suggested that drought stimulated *S. spontaneum* sterols to protect cells and maintain normal photosynthesis. Similarly, transcriptome analysis by Wang et al.^27^ revealed that the *phosphoesterase* gene was up-regulated and played an active role in sugarcane drought response.

Plant protein kinases such as mitogen-activated protein kinase (MAPK) are involved in the hormone signaling of plants^62^. When plants are subjected to abiotic stress, related MAPK family genes' transcription factors are up-regulated, thereby enhancing the plant resistance^63^. Herein, the pathway annotation of the DEGs revealed that the transcription level of protein kinase-related genes, including MAPK, was up-regulated (Supplementary Table S1). This finding indicates that the MAPK family genes play an essential role in enhancing the drought resistance ability of *S. spontaneum.* Plants accumulate large amounts of trehalose under stress. Increased trehalose content improves plants' ability to withstand abiotic environments such as low temperature, drought, high temperature, and high salt content^64–66^. Herein, the functional genes of trehalose phosphatase (Unigene2276, Unigene23524, and Unigene23525) were up-regulated, which possibly enhanced the drought tolerance ability of *S. spontaneum*. Notably, not all genes conducive to plant stress resistance are up-regulated. For example, 4-coumarate-CoA ligase is a key enzyme required to produce various natural products. Down-regulation of this gene leads to a decrease in lignin and flavonoids production^67^. Flavonoid compounds have a vital antioxidant function that improves the stress resistance of plants^68^. However, the transcription levels of flavone and flavonol biosynthesis gene and 4-coumarate-CoA ligase gene (Unigene37048) in *S. spontaneum* were down-regulated under drought stress. The down-regulation caused the plants to reduce lignin and flavonoids production in the absence of water, thereby causing various diseases.

Plant responses begin with stress recognition at the cellular level through activation of signal transduction pathways. Herein, 47 DEGs were involved in plant hormone signal transduction pathways. Previous studies postulate pathways based on PYR/RCAR ABA receptors, protein phosphatase 2C (PP2Cs), and serine/threonine-protein kinase 2 (SnRK2s) form the primary basis of early ABA signaling module. Generally, the PYR/RCARs act as ABA receptors, the PP2Cs act as negative regulators of the pathway, and SnRK2s act as positive regulators of downstream signaling^69,70^. Pre-existing PP2Cs appear to repress the ABA signaling pathways by inactivating SnRK2s in the absence of ABA under normal conditions. ABA production increases under water stress, thereby causing ABA-bound PYR/RCARs to interact with PP2Cs and inhibit phosphatase activity. These occurrences promote SnRK2 activation and the phosphorylation of target proteins^70,71^. In this study, the expression of eleven PP2Cs genes (Unigene26830, Unigene3354, Unigene22407, Unigene22408, Unigene26313, Unigene22406, Unigene26314, CL4935.Contig6, CL9703.Contig1, Unigene20159, Unigene10307) and three SnRK2 genes (Unigene20990, Unigene22490, Unigene22488) was significantly induced by water stress. The genes were correlated with maximum enzyme activity under water stress ^69,70^. Besides, the bHLH-type transcription factor (Unigene17371), which positively regulates ABA response in *Arabidopsis*^72^*,* was significantly induced by water stress. Three indole-3-acetic acid-amido synthetase genes (Unigene45198, Unigene5209, and Unigene47133) were also up-regulated, similar to the IAA content increment under water stress.

Stress resistance in maize is closely related to the degree of gene expression of expansion proteins genes *Exp1*, *Exp5*, and *ExpB8* in the apical elongation region^73^. Accumulation and expression of *OsEXP2* in rice's root tip maintains continuous extension of the root tip cell wall and plays a positive role in plant growth under arid environments^74^. Herein, drought conditions failed to induce expression of the extended protein gene (CL5005.Contig1) in *S. spontaneum.* Up-regulation of the extended protein gene after rehydration was conducive to root growth in *S. spontaneum* and then enhanced its water absorption ability to maintain normal growth (Supplementary Table S1). Plant LRR type receptor-like serine/threonine-protein kinase is an essential carrier of signal transmission. It detects external environmental stresses, thus playing an important regulatory role in plant response to various stresses^75^. Lee et al. reported that the LRR type receptor-like protein kinase gene *OsRLK1* of rice was induced and expressed by low temperature and salt stress^76^. Similarly, Jung et al. found that expression of *CaLRR1* in pepper was induced by anthracnose pathogens and other abiotic stresses such as high salt content, ABA, and mechanical damage^77^. Herein, the LRR-type receptor kinase genes (Unigene60151, Unigene34533, CL5366.Contig1) were up-regulated after rehydration, thus helping plants to recover quickly after a water loss. Other beneficial genes that enhance plant drought resistance were also up and down-regulated under water stress. For instance, the proline-rich protein gene is involved in plant defense response to abiotic stresses. Expression of the pigeon pea (*CcHyPRP*) gene improves transgenic *Arabidopsis*' resistance to drought, high salt content, and high temperature^78^. In this study, the proline-rich protein gene (Unigene20869) was down-regulated under stress but up-regulated after rehydration.

After re-watering, the distribution of DEGs was significantly correlated with catalytic activity (58) and binding (67) functions. After rehydration, there were 259 DEGs, accounting for only 19% of the total DEGs (1355) under water stress. This finding implies that 81% of the total DEGs and 97% (1310) of DEGs under drought conditions returned to normal expression levels after rehydration. The newly added DEGs accounted for only 16% under drought conditions during re-watering. This phenomenon strongly suggests that *S. spontaneum* could induce a memory response after plant re-watering to restore normal growth. Similar findings were obtained in rice^79^, potato^80^, and poplar^81^. Nonetheless, the expression of some genes did not differ between the controls and stressed plants but was only up-or down-regulated after re-watering. They included genes encoding for the nucleotide-binding site (NBS)-leucine-rich repeat (LRR) protein, wall-associated kinases, cysteine proteinase, and lecithin-cholesterol acyltransferase (LCAT) (Supplementary Table S1). The NBS-LRR protein plays an essential role in plant disease resistance signal transduction^82^. Herein, the *NBS-LRR* gene (Unigene54434) was only up-regulated after re-watering, indicating that *S. spontaneum* had a reduced risk of disease attack after re-watering. The *WAK* gene plays an important role in plant innate immunity. Induced expression of the *WAK* gene enhances plant resistance to fungal pathogens^83,84^ and affects the up-regulation of ethylene and methyl jasmonate signaling molecules^85^. Induction of cysteine protease in drought-stressed plants is common, but the induction level is negatively correlated with drought resistance^86^. Besides, cysteine proteases are also involved in plant defense mechanisms against insects^87^. Herein, the expression of the *LCAT* gene (Unigene6969) was down-regulated 7.88 folds after rehydration. Chen et al.^88^ reported that high expression of the *LCAT* gene in *Arabidopsis* roots and siliques plays an important role in root development and/or lipid metabolism. There are currently only a few reports on the role of the *LCAT* gene in plants despite numerous studies regarding its role in human medicine. It is, therefore, important to further explore the value of this gene in plants.

## Conclusions

*S. spontaneum* GXS87-16 has potent ability to withstand water stress through a quick accumulation of osmoprotectant (proline) and plant hormones (ABA, SA, and IAA). Reduction of GA_3_, high antioxidase activity (CAT, SOD, APX, and GR), and quick recovery after re-watering also promote its drought tolerance ability. Transcriptome profiling indicated that the DEGs under drought stress were enriched in plant protein kinases and plant hormone signal transduction pathways responsible for drought tolerance. The findings of this study reveal the most critical pathways associated with drought stress in *S. spontaneum* and will help characterize the pathways and guide molecular breeding to develop drought-tolerant sugarcane varieties.

## Materials and methods

### Plant materials and drought stress treatment

The *S. spontaneum* GXS87-16 used in this experiment was obtained from the Sugarcane Research Institute of the Guangxi Academy of Agricultural Sciences. Single bud sets of GXS87-16 were initially raised through sand culture techniques and maintained for 40 days. The 40-day-old seedlings were transplanted into pots (28 cm diameter × 35 cm height) containing 25 kg of soil mixture [clay/organic fertilizer/sand in the ratio of 80:10:10 (w/w), with a basal dose of NPK fertilizer (25 g N + 2 g P + 20 g K pot^-1^)]. Irrigation was done daily under greenhouse conditions. Drought stress was induced during plant growth during the elongation stage (after five months of plant growth). It is the most important period for *S. spontaneum* production and the critical water demand period^89^. Drought stress was induced by halting watering. The total soil moisture content was determined using the gravimetric method (soil moisture% (dw) = 100 × [(fresh weight-dry weight)/dry weight]). The total water content in control and drought-treated pots was maintained at 18 ± 2% and 9 ± 2%, respectively. Water stressed leaf samples were collected on the 3^rd^, 6^th^, and 9^th^ day after watering was stopped (denoted as WS-3, WS-6, and WS-9) to represent mild, moderate, and severe water deficit conditions, respectively. The re-watered leaf samples (denoted as RW) were collected on the 4^th^ day after re-watering the severely water-deficient plants. The top visible dewlap leaf samples were detached from six plants for each treatment (two plants combined to form one biological replicate—totaling three biological replicates for each treatment) and frozen in liquid nitrogen for further use. Analysis of physiological and biochemical parameters was done in triplicate for all samples.

### Determination of RWC, H_2_O_2_, proline and MDA contents

Leaf relative water content (RWC) was gravimetrically measured as described by Silva et al.^90^. Three leaf disks (1.3 cm diameter each) were collected per plant using a cork borer, immediately sealed in glass vials, and transported to the lab in an ice-cooled chest. Their fresh weights (FW) were determined within 1 h after excision. Their turgid weight (TW) was obtained after rehydration in deionized water for 24 h at room temperature. The disks were subsequently dried in an oven at 80 °C for 48 h, and their dry weights (DW) were determined. RWC was then determined using the formula: RWC = (FW-DW) / (TW-DW) ×100%.

The H_2_O_2_ content was determined using a modified method described by Gniazdowska et al.^91^. Fresh leaf samples (0.5 g) were homogenized with 5 mL of 3% (*w/v*) trichloroacetic acid (TCA) in an ice bath followed by centrifugation of the homogenate at 12,000 ×*g* for 15 min. This step was followed by mixing 1 mL of supernatant with 1 mL of 100 mM potassium phosphate buffer (pH 7.0) and 2 mL of 1 M KI. The H_2_O_2_ content was then measured at 390 nm and calculated using a standard curve ranging between 10 and 100 µM.

The proline (PRO) content was quantified as described by Bates et al.^92^. Leaf samples (0.5 g) were ground to powder using liquid nitrogen then homogenized with 10 mL of 3% (*w/v*) sulfosalicylic acid. The homogenate was then centrifuged at 1000 × *g* for 5 min at 4 °C and 2 mL of the supernatant mixed with 2 mL of glacial acetic acid and 3 mL of acid ninhydrin. The mixtures were then incubated at 100 °C for 1 h, cooled, and 5 mL toluene added. The mixtures were then centrifuged at 1000 ×*g* for 5 min at 4 °C, and the absorbance of the supernatant was determined at 520 nm.

Malondialdehyde (MDA) contents were determined using a method described by Heath and Packer^93^. The frozen samples (0.5 g) were homogenized in 10 mL of 10% (*w/v*) trichloroacetic acid (TCA), and the mixture centrifuged at 10,000 ×*g* for 10 min. The supernatant (2 mL) was mixed with 2 mL of 0.6% (*w/v*) thiobarbituric acid (TBA) or 2 mL of distilled water for the control and then heated in a boiling water bath for 15 min. The mixture was then cooled in an ice bath to stop the reaction. It was then centrifuged at 10,000 ×*g* for 10 min at 4 °C, and the supernatant's absorbance measured at 450 nm, 532 nm, and 600 nm.

### Measurement of antioxidant enzyme activity

Fresh leaf samples (0.5 g) were ground to powder using liquid nitrogen and homogenized with 5 mL phosphate buffer (10 mM, pH 7.4). The homogenate was then centrifuged at 8,000 ×*g* for 15 min at 4 °C, and the supernatant was used to measure the activity of various antioxidant enzymes. The catalase (CAT, BC0200), superoxide dismutase (SOD, BC0170), ascorbate peroxidase (APX, BC0220), and glutathione reductase (GR, BC1165) activities were measured using ELISA kits from Beijing Solarbio Science & Technology Co., Ltd. (Beijing, China) following the manufacturer’s instructions.

### Measurement of endogenous plant hormones

Endogenous hormone content was measured using an ELISA kit sourced from Shanghai Jing Kang Bioengineering Co., Ltd. (Shanghai, China). Samples were prepared as described by Zeng et al.^94^. Approximately 0.5 g of fresh samples were ground in an ice bath using 5 mL of 80% methanol as the extraction solution. The extracts were incubated at 4 °C overnight and then centrifuged at 12,000 ×*g* for 15 min at 4 °C*.* The supernatant was then passed through a Chromosep C_18_ column prewashed with 10 ml of 100% methanol and 5 mL of 80% methanol. The combined supernatant was used to measure the abscisic acid (ABA, JLC11479), salicylic acid (SA, JLC11446), indoleacetic acid (IAA, JLC11517), and gibberellin (GA_3_, JLC11814) concentrations following the manufacturer’s instructions.

### RNA extraction and RNA-seq library construction for Illumina sequencing

The control, WS-9 and RW leaf samples (three biological replicates for each, totaling nine RNA-seq libraries) were used for transcriptome analysis. Total RNA was extracted using the Trizol RNA extraction kit (Invitrogen, USA). RNA quality was determined using 1.2% agarose gels and a NanoPhotometer micro-spectrophotometer. Quantification of the RNA was done using the Qubit RNA analysis kit (Life Technologies, USA) and its integrity using the RNA Nano 6000 assay kit in the Agilent Bioanalyzer 2100 system (Agilent Technologies, USA).

RNA-seq libraries were generated using NEB Next Ultra RNA Library Prep Kit for Illumina (NEB, USA) following the manufacturer’s protocol. Briefly, mRNA was purified from the total RNA using magnetic beads carrying Oligo (dT) and then cleaved into small fragments using fragmentation buffer. First-strand cDNA was synthesized using random hexamer primer followed by second-strand cDNA synthesis using RNase H and DNA polymerase I. Double-stranded cDNA was then subjected to terminal repair, A-tailing, sequence adaptor ligation, fragment screening, PCR amplification, PCR product purification, and library quality assessment. The libraries were then sequenced in an Illumina HiSeq4000 platform using the paired-end technology developed by BGI Genomics Co. Ltd. (Shenzhen, China).

### Data filtering, gene functional annotation and differentially expressed genes screening

Raw reads were generated through the Illumina data processing pipeline. Clean reads were obtained by removing low-quality bases, empty reads, and adaptor sequences at the 3′ end from the raw reads. The Q20, GC content, and sequence duplication level of the clean data were assessed using the Trinity software^95^. Functional gene annotations were collected for transcript sequences ≥150 bp by searching against the NCBI protein database using the BLASTX tool (e-value <10^-5^). The GO functional annotations were searched through GO analysis (http://geneontology.org/) (e-value <10^-5^ cut-off), and the results plotted using the annotation numbers in the WEGO analysis (http://wego.genomics.org.cn/cgi-bin/wego/index.pl). Gene pathway annotations were performed using the KAAS tool (http://www.genome.jp/tools/kaas/) (e-value <10^-5^). For gene expression analysis, fragments per kilobase of exon per million fragments mapped read (FPKM) was calculated at the expression level^96^. Differentially Expressed Genes (DEGs) were calculated in FPKM by comparing the gene expression of different treatments using the DESeq R package^97^. The minimum false discovery rate (FDR) was calculated, and the DEGs were selected at FDR (*q*) <0.05 to avoid false positives.

### Validation of RNA-seq data using qRT-PCR

Fifteen candidate genes related to resistance were randomly selected to determine the accuracy of the DEGs-based gene expression using qRT-PCR. The specific primers of the candidate genes and reference gene GAPDH (Glyceraldehyde-3-phosphate dehydrogenase, EF189713) were designed using the Primer Premier 5.0 software (Supplementary Table S2). Total RNA (1 µg) was used to synthesize the first-strand cDNA using the PrimeScript RT Master Mix (TaKaRa, Japan) following the manufacturer’s instructions. Three biological and technical replicates were processed for the qRT-PCR assays on an ABI 7500 Real-Time PCR System. The assay was performed in 20 μL reaction volumes containing 10 µL SYBRGREEN I Master Mix (TaKaRa, Japan), 2μL cDNA, 1μL of each primer (4 μM), and 6μL RNase-free sterile water. The PCR conditions were: initial denaturation at 95 °C, for 60 s, followed by 40 cycles of denaturation and annealing at 95 °C for 10 s and 60 °C for 20 s, respectively. Each reaction's specificity was determined through the melting curve analysis, and the relative expression levels calculated using the 2^−ΔΔCt^ method^98^.

### Data analysis

Data were subjected to an analysis of variance (ANOVA) and mean separation was performed using the least significance difference (LSD, *P* < 0.05) procedures of the SPSS statistical package (SPSS Student Version 15.0), and heatmap generated by using ClustVis online tool (https://biit.cs.ut.ee/clustvis/)^99^.

## Supplementary Information

Supplementary Information

## Data Availability

The Illumina raw reads of samples at different time points were deposited in NCBI BIO PROJECT with accession number PRJNA684430.
