# Supplementary material for: Physiological changes and transcriptome profiling in Saccharum spontaneum L. leaf under water stress and re-watering conditions
Source: Sci Rep. 2021 Mar 9;11:5525. doi: 10.1038/s41598-021-85072-1 (PMC7943799; doi:10.1038/s41598-021-85072-1)
Supplement: Supplementary file 1 — Supplementary Information [file 41598_2021_85072_MOESM1_ESM.pdf]

Physiological Changes and Transcriptome Profiling in *Saccharum spontaneum* L. Leaf under Water Stress and Re-watering Conditions

Changning Li[1#], Zhen Wang[2#], Qian Nong[3]*, Li Lin[1], Jinlan Xie[1], Zhanghong Mo[1], Xing Huang[1], Xiupeng Song[1], Mukesh Kumar Malviya[1], Manoj Kumar Solanki[4] & Yangrui Li[1]*

[1]Key Laboratory of Sugarcane Biotechnology and Genetic Improvement (Guangxi), Ministry of Agriculture / Guangxi Key Laboratory of Sugarcane Genetic Improvement, Nanning 530007, China.

[2]College of Biology and Pharmacy, Yulin Normal University, Yulin 537000, China.

[3]Plant Protection Research Institute, Guangxi Academy of Agricultural Sciences, Nanning 530007, China.

[4]Department of Food Quality & Safety, The Volcani Center, Institute for Post-harvest and Food Sciences, Agricultural Research Organization, Rishon LeZion, Israel.

*Correspondence and requests for materials should be addressed to Q. N. (email: nongqian@126.com) or Y. L. (email: liyr@gxaas.net)

[#]These authors contributed equally to this work

Supplementary information

Figure S1 Expression patterns of 45 overlapped genes under WS-9 and RW treatments

Table S1 Pathway category details of stress-responsive genes

Table S2 Validation of selected candidate genes using real time-PCR

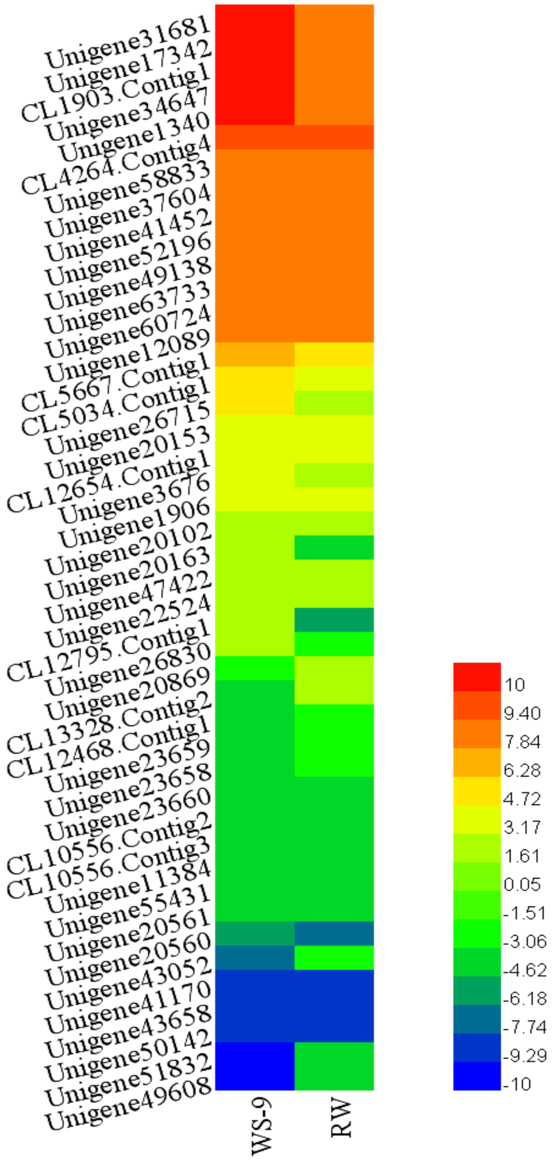

Fig. S1 Expression patterns of 45 overlapped genes under WS-9 and RW treatments. Leaf samples were collected at WS-9 and RW conditions, which corresponded to stop watering 9th days and 4th days after re-watering, respectively. The color bar at the right side represents log2 expression values: Blue represents low expression, green medium expression and red high expression.

Table S1 Pathway category details of stress-responsive genes

Gene ID	Description	Blast species	e_value	WS-9_FC	RW_FC
**RNA transport**					
Unigene37048	4-coumarate-CoA ligase	Zea mays	3.60E-09	-9.09	–
CL11154.Contig1	Thaumatin-like protein	Pyrus pyrifolia	4.90E-64	-5.59	–
Unigene23042	DUF231 domain containing family protein	Zea mays	0.00E+00	-4.38	–
Unigene713	phototropic-responsive NPH3 family protein	Zea mays	1.10E-54	-4.14	–
CL1882.Contig2	myosin-9	Sorghum bicolor	2.00E-115	-3.83	–
Unigene23962	protein FAF-like	Sorghum bicolor	0.00E+00	-3.19	–
CL5236.Contig2	protein targeted either to mitochondria or chloroplast proteins	Sorghum bicolor	9.40E-99	-3.17	–
Unigene1467	lichenase-2	Zea mays	0.00E+00	-3.14	–
Unigene18958	myosin-9	Sorghum bicolor	3.00E-64	-2.72	–
Unigene2660	DUF1645 family protein	Zea mays	1.00E-27	-2.52	–
CL7372.Contig1	zinc finger CCCH domain-containing protein	Zea mays	0.00E+00	-2.51	–
Unigene20594	chaperone protein dnaJ 20	Zea mays	2.00E-91	-2.38	–
Unigene1680	zinc finger protein	Oryza brachyantha	9.60E-52	2.08	–
CL177.Contig1	AP2-associated protein kinase	Actinidia chinensis	4.00E-16	2.28	–
Unigene2767	nuclear factor YB2	Sorghum bicolor	2.50E-80	2.36	–
Unigene25500	Oxysterol-binding protein-related protein like	Actinidia chinensis	1.00E-39	2.53	–
Unigene21978	vegetative cell wall protein gp1	Setaria italica	1.00E-132	2.57	–
Unigene24010	FAD-dependent urate hydroxylase	Acinetobacter baylyi	4.00E-06	2.59	–
Unigene14105	protein NUCLEAR FUSION DEFECTIVE 4	Sorghum bicolor	0.00E+00	2.62	–
Unigene2182	transcription factor bHLH041	Arabidopsis thaliana	1.50E-19	2.71	–
Unigene23050	Zeamatin	Zea mays	3.20E-121	2.96	–
CL9281.Contig1	transferase family protein	Zea mays	0.00E+00	3.00	–
Unigene31996	non-lysosomal glucosylceramidase	Sorghum bicolor	0.00E+00	3.02	–
Unigene21916	Pathogenesis-related protein	Juniperus ashei	1.50E-57	3.04	–
Unigene1460	F-box protein	Sorghum bicolor	0.00E+00	3.08	–
Unigene1906	transposon protein CACTA, En/Spm sub-class	Zea mays	0.00E+00	3.27	3.21
Unigene909	Protein kinase domain superfamily protein	Zea mays	1.00E-48	3.28	–
Unigene1583	galactinol synthase	Setaria italica	1.00E-65	3.37	–
Unigene20878	DEAD-box ATP-dependent RNA helicase	Oryza sativa	6.00E-51	3.39	–
CL2470.Contig1	glucan endo-1,3-beta-glucosidase	Zea mays	3.64E-171	3.47	–
Unigene28135	GEM-like protein 5	Arabidopsis thaliana	6.70E-79	3.49	–
CL6955.Contig1	ocs element-binding factor	Setaria italica	3.52E-78	3.51	–
CL12417.Contig2	GSDL-motif lipase	Zea mays	0.00E+00	3.57	–
Unigene24872	ricin B-like lectin	Sorghum bicolor	0.00E+00	3.67	–
CL13350.Contig1	CHP-rich zinc finger protein-like	Oryza sativa	4.00E-35	3.72	–
CL9329.Contig1	BAG family molecular chaperone regulator 6	Zea mays	0.00E+00	3.76	–
CL10903.Contig1	dehydrin	Sorghum bicolor	5.60E-20	3.94	–
CL8702.Contig1	chitinase	Saccharum officinarum	4.30E-148	4.02	–
Unigene17998	hepatoma-derived growth factor-related protein	Sorghum bicolor	0.00E+00	4.31	–
Unigene28610	glucan endo-1,3-beta-glucosidase 1-like	Zea mays	0.00E+00	4.45	–
CL13599.Contig1	beta-1,3-glucanase	Sorghum bicolor	0.00E+00	4.73	–
Unigene43577	DC1 domain-containing protein	Corchorus capsularis	7.00E-27	4.87	–
Unigene26573	2-oxoglutarate (2OG) and Fe(II)-dependent oxygenase protein	Zea mays	0.00E+00	5.02	–
Unigene23943	disulfide isomerase	Saccharum officinarum	1.30E-101	5.40	–
CL5895.Contig2	sulfate transporter	Sorghum bicolor	0.00E+00	5.57	–
Unigene23944	disulfide isomerase	Saccharum officinarum	1.20E-115	5.60	–
Unigene4586	Universal stress protein A-like protein	Arabidopsis thaliana	1.30E-06	5.87	–
Unigene3564	gibberellin receptor GID1L2	Zea mays	0.00E+00	6.25	–
Unigene23961	mitogen-activated protein kinase kinase kinase	Setaria italica	1.00E-103	6.52	–
Unigene20212	hepatoma-derived growth factor-related protein	Sorghum bicolor	7.00E-09	7.16	–
CL3146.Contig1	Low temperature-induced protein	Hordeum vulgare	9.40E-14	7.53	–

Table S1 (Continued)

Gene ID	Description	Blast species	e_value	WS-9_FC	RW_FC
CL9922.Contig2	methyltransferase	Oryza sativa	5.70E-25	7.77	–
Unigene39496	protein YLS9-like	Zea mays	2.30E-68	8.02	–
Unigene33764	retrotransposon protein	Oryza sativa	2.30E-54	8.10	–
CL7484.Contig2	plant/MUD21-2 protein	Zea mays	5.00E-171	8.18	–
Unigene17376	L-gulonolactone oxidase	Zea mays	4.50E-99	8.30	–
CL8801.Contig1	plant/MUD21-2 protein	Zea mays	5.00E-171	8.34	–
Unigene35089	polyphenol oxidase	Sorghum bicolor	1.70E-37	8.38	–
Unigene8088	calcium-binding protein	Sorghum bicolor	5.00E-67	8.45	–
Unigene38023	cation/H(+) antiporter 28-like	Zea mays	6.40E-67	8.57	–
CL7354.Contig1	lecithin-cholesterol acyltransferase	Setaria italica	2.00E-12	8.64	–
Unigene12127	translation initiation factor IF-2	Zea mays	1.00E-83	8.64	–
Unigene15413	DUF679 domain membrane protein	Zea mays	4.00E-103	8.72	–
Unigene65830	CCT motif family protein	Zea mays	6.00E-61	8.73	–
Unigene15349	L-gulonolactone oxidase-like	Zea mays	1.20E-101	9.00	–
Unigene36989	carboxylesterase	Setaria italica	1.30E-44	9.09	–
Unigene22689	ribonuclease H-like domain-containing protein	Medicago truncatula	2.00E-37	9.24	–
Unigene35215	protein EMSY-LIKE 3	Sorghum bicolor	5.00E-52	9.73	–
Unigene19047	verprolin-like	Panicum hallii	4.00E-12	9.94	–
Unigene27617	Expansin	Oryza sativa	1.30E-115	–	5.92
CL5005.Contig1	expansin-B3-like	Zea mays	2.00E-135	–	3.55
**mRNA surveillance pathway**					
Unigene37048	4-coumarate-CoA ligase	Zea mays	3.60E-09	-9.09	–
CL11154.Contig1	Thaumatin-like protein	Pyrus pyrifolia	4.90E-64	-5.59	–
Unigene20561	flowering promoting factor-like	Zea mays	4.49E-152	-4.61	-4.58
Unigene23042	DUF231 domain containing family protein	Zea mays	0.00E+00	-4.38	–
Unigene713	phototropic-responsive NPH3 family protein	Zea mays	1.10E-54	-4.14	–
Unigene23962	protein FAF-like	Sorghum bicolor	0.00E+00	-3.19	–
CL5236.Contig2	protein targeted either to mitochondria or chloroplast proteins	Sorghum bicolor	9.40E-99	-3.17	–
Unigene1467	lichenase-2	Zea mays	0.00E+00	-3.14	–
Unigene18958	myosin-9	Sorghum bicolor	3.00E-64	-2.72	–
Unigene2660	DUF1645 family protein	Zea mays	1.00E-27	-2.52	–
CL7372.Contig1	zinc finger CCCH domain-containing protein	Zea mays	0.00E+00	-2.51	–
Unigene3157	Flowering-promoting factor 1-like protein 5	Oryza sativa	3.30E-37	-2.45	–
Unigene20594	chaperone protein dnaJ 20	Zea mays	2.00E-91	-2.38	–
Unigene1680	zinc finger protein	Oryza brachyantha	9.60E-52	2.08	–
CL177.Contig1	AP2-associated protein kinase	Actinidia chinensis	4.00E-16	2.28	–
Unigene2767	nuclear factor YB2	Sorghum bicolor	2.50E-80	2.36	–
Unigene25500	Oxysterol-binding protein-related protein like	Actinidia chinensis	1.00E-39	2.53	–
Unigene21978	vegetative cell wall protein gp1	Setaria italica	1.00E-132	2.57	–
Unigene24010	FAD-dependent urate hydroxylase	Acinetobacter baylyi	4.00E-06	2.59	–
Unigene14105	protein NUCLEAR FUSION DEFECTIVE 4	Sorghum bicolor	0.00E+00	2.62	–
Unigene2182	transcription factor bHLH041	Arabidopsis thaliana	1.50E-19	2.71	–
Unigene23050	Zeamatin	Zea mays	3.20E-121	2.96	–
CL9281.Contig1	transferase family protein	Zea mays	0.00E+00	3.00	–
Unigene31996	non-lysosomal glucosylceramidase	Sorghum bicolor	0.00E+00	3.02	–
Unigene21916	Pathogenesis-related protein	Juniperus ashei	1.50E-57	3.04	–
Unigene19686	loricrin	Saccharum officinarum	6.90E-17	3.05	–
Unigene1460	F-box protein	Arabidopsis thaliana	3.20E-90	3.08	–
Unigene909	Protein kinase domain superfamily protein	Zea mays	1.00E-48	3.28	–
Unigene1583	galactinol synthase	Setaria italica	1.00E-65	3.37	–
Unigene20878	DEAD-box ATP-dependent RNA helicase	Oryza sativa	6.00E-51	3.39	–
Unigene31211	eukaryotic peptide chain release factor subunit	Oryza brachyantha	4.40E-223	3.42	–

Table S1 (Continued)

Gene ID	Description	Blast species	e_value	WS-9_FC	RW_FC
CL2470.Contig1	glucan endo-1,3-beta-glucosidase	Zea mays	3.64E-171	3.47	–
Unigene28135	GEM-like protein 5	Arabidopsis thaliana	6.70E-79	3.49	–
CL6955.Contig1	ocs element-binding factor	Setaria italica	3.52E-78	3.51	–
CL10903.Contig1	dehydrin	Sorghum bicolor	5.60E-20	3.94	–
Unigene17998	hepatoma-derived growth factor-related protein	Sorghum bicolor	0.00E+00	4.31	–
Unigene28610	glucan endo-1,3-beta-glucosidase 1-like	Zea mays	0.00E+00	4.45	–
CL13599.Contig1	beta-1,3-glucanase	Sorghum bicolor	0.00E+00	4.73	–
Unigene43577	DC1 domain-containing protein	Corchorus capsularis	7.00E-27	4.87	–
Unigene26573	2-oxoglutarate (2OG) and Fe(II)-dependent oxygenase protein	Zea mays	0.00E+00	5.02	–
Unigene23943	disulfide isomerase	Saccharum officinarum	1.30E-101	5.40	–
CL5895.Contig2	sulfate transporter	Zea mays	1.70E-232	5.57	–
Unigene23944	disulfide isomerase	Saccharum officinarum	1.20E-115	5.60	–
Unigene4586	Universal stress protein A-like protein	Arabidopsis thaliana	1.30E-06	5.87	–
Unigene3564	gibberellin receptor GID1L2	Zea mays	0.00E+00	6.25	–
Unigene23961	mitogen-activated protein kinase kinase kinase	Setaria italica	1.00E-103	6.52	–
Unigene20212	hepatoma-derived growth factor-related protein	Sorghum bicolor	7.00E-09	7.16	–
CL3146.Contig1	Low temperature-induced protein	Hordeum vulgare	9.40E-14	7.53	–
CL9922.Contig2	methyltransferase	Oryza sativa	5.70E-25	7.77	–
Unigene39496	protein YLS9-like	Zea mays	2.30E-68	8.02	–
Unigene33764	retrotransposon protein	Oryza sativa	2.30E-54	8.10	–
CL7484.Contig2	plant/MUD21-2 protein	Zea mays	5.00E-171	8.18	–
Unigene17376	L-gulonolactone oxidase	Zea mays	4.50E-99	8.30	–
Unigene35089	polyphenol oxidase	Sorghum bicolor	1.70E-37	8.38	–
Unigene8088	calcium-binding protein	Sorghum bicolor	5.00E-67	8.45	–
Unigene38023	cation/H(+) antiporter 28-like	Zea mays	6.40E-67	8.57	–
CL7354.Contig1	lecithin-cholesterol acyltransferase	Setaria italica	2.00E-12	8.64	–
Unigene12127	translation initiation factor IF-2	Zea mays	1.00E-83	8.64	–
Unigene15413	DUF679 domain membrane protein	Zea mays	4.00E-103	8.72	–
Unigene56538	RNA-binding protein	Arabidopsis thaliana	6.40E-08	8.88	–
Unigene15349	L-gulonolactone oxidase-like	Zea mays	1.20E-101	9.00	–
Unigene36989	carboxylesterase	Setaria italica	1.30E-44	9.09	–
Unigene22689	ribonuclease H-like domain-containing protein	Medicago truncatula	2.00E-37	9.24	–
Unigene19047	verprolin-like	Panicum hallii	4.00E-12	9.94	–
CL5005.Contig1	expansin-B3-like	Zea mays	2.00E-135	–	3.55
**Plant hormone signal transduction**					
Unigene8695	L-type lectin-domain containing receptor kinase	Arabidopsis thaliana	2.50E-17	-7.94	–
Unigene26133	Auxin-responsive protein IAA31	Oryza sativa	3.10E-15	-3.04	–
Unigene27079	Auxin response factor 8	Oryza sativa	2.50E-189	-2.74	–
CL1845.Contig3	IAA17-auxin-responsive Aux/IAA family member	Zea mays	1.00E-93	-2.66	–
CL1845.Contig2	Auxin-responsive protein IAA3	Oryza sativa	2.70E-43	-2.40	–
Unigene26830	protein phosphatase 2C	Oryza sativa	6.90E-118	2.15	-2.32
Unigene3354	protein phosphatase 2C	Oryza sativa	2.00E-126	2.24	–
Unigene18758	histidine-containing phosphotransfer protein	Zea mays	0.00E+00	2.50	–
Unigene17650	Cysteine-rich receptor-like protein kinase	Arabidopsis thaliana	1.90E-11	2.51	–
CL7470.Contig1	STRUBBELIG family receptor protein kinase	Zea mays	2.70E-180	2.81	–
Unigene22407	protein phosphatase 2C	Setaria italica	3.71E-36	2.82	–
CL367.Contig1	atypical receptor-like kinase MARK	Zea mays	0.00E+00	2.96	–
Unigene22408	protein phosphatase 2C	Setaria italica	4.00E-56	3.06	–
Unigene20990	Serine/threonine-protein kinase HT1	Arabidopsis thaliana	3.30E-81	3.13	–
Unigene26313	protein phosphatase 2C	Oryza sativa	2.30E-64	3.21	–
Unigene22490	LRR receptor-like serine/threonine-protein kinase	Setaria italica	5.63E-129	3.28	–
Unigene22406	protein phosphatase 2C	Setaria italica	1.47E-155	3.31	–

Table S1 (Continued)

Gene ID	Description	Blast species	e_value	WS-9_FC	RW_FC
Unigene22488	LRR receptor-like serine/threonine-protein kinase	Setaria italica	2.40E-31	3.40	–
Unigene34056	receptor protein kinase ZmPK1	Zea mays	6.80E-38	3.42	–
Unigene26314	protein phosphatase 2C	Oryza sativa	7.20E-26	3.47	–
Unigene22005	Protein STRUBBELIG-RECEPTOR FAMILY 6	Arabidopsis thaliana	1.10E-68	3.49	–
CL11969.Contig1	probable L-type lectin-domain containing receptor kinase	Zea mays	3.20E-147	3.67	–
Unigene17371	transcription factor BHLH148	Sorghum bicolor	0.00E+00	3.93	–
CL4935.Contig6	protein phosphatase 2C	Setaria italica	2.31E-104	3.96	–
Unigene15066	Transcription factor ABORTED MICROSPORES	Arabidopsis thaliana	7.70E-18	3.97	–
CL4655.Contig1	receptor-like protein kinase	Zea mays	3.46E-128	4.31	–
CL3365.Contig3	Protein STRUBBELIG-RECEPTOR FAMILY	Arabidopsis thaliana	4.20E-40	4.44	–
Unigene21956	inactive receptor kinase RLK902	Arabidopsis thaliana	6.70E-37	4.44	–
CL12929.Contig1	Protein TIFY 11c	Oryza sativa	6.60E-40	4.69	–
Unigene17931	pathogenesis-related protein	Setaria italica	3.20E-18	4.71	–
Unigene1014	pathogenesis-related protein 1	Zea mays	1.10E-13	4.80	–
Unigene22447	2C-type protein phosphatase protein	Zea mays	0.00E+00	5.45	–
CL9703.Contig1	protein phosphatase 2C	Setaria italica	0.00E+00	5.71	–
Unigene20159	protein phosphatase 2C 8	Oryza sativa	1.00E-90	5.93	–
CL5034.Contig1	pathogenesis-related protein	Zea mays	3.40E-42	6.09	3.87
Unigene3564	gibberellin receptor GID1L2	Zea mays	0.00E+00	6.25	–
CL11032.Contig1	L-type lectin-domain containing receptor kinase	Brachypodium distachyo	7.80E-77	7.03	–
Unigene52816	scarecrow-like protein	Zea mays	6.12E-73	8.06	–
Unigene34151	ZIM transcription factor	Zea mays	8.00E-35	8.12	–
Unigene45198	indole-3-acetic acid-amido synthetase	Oryza sativa	4.20E-40	8.12	–
Unigene5209	indole-3-acetic acid-amido synthetase	Oryza sativa	5.50E-08	8.65	–
Unigene47133	indole-3-acetic acid-amido synthetase	Oryza sativa	8.50E-59	8.83	–
Unigene36989	carboxylesterase	Setaria italica	1.30E-44	9.09	–
Unigene10307	protein phosphatase 2C 8	Zea mays	8.92E-120	9.22	–
Unigene17932	Pathogenesis-related protein PRMS	Zea mays	4.70E-32	9.87	–
Unigene20764	L-type lectin-domain containing receptor kinase	Arabidopsis thaliana	1.10E-138	10.25	–
CL3750.Contig2	wall-associated receptor kinase	Arabidopsis thaliana	1.10E-32	–	5.82
**Plant-pathogen interaction**					
Unigene8695	L-type lectin-domain containing receptor kinase	Arabidopsis thaliana	2.50E-17	-7.94	–
CL5816.Contig1	Phosphoenolpyruvate carboxylase kinase	Arabidopsis thaliana	8.80E-61	2.16	–
CL11545.Contig1	WRKY transcription factor	Zea mays	2.80E-42	2.22	–
CL13529.Contig1	LRR receptor-like serine/threonine-protein kinase	Arabidopsis thaliana	1.80E-191	2.35	–
Unigene29535	calcium-binding protein CML16	Oryza sativa	2.60E-57	2.39	–
Unigene17650	Cysteine-rich receptor-like protein kinase	Arabidopsis thaliana	1.90E-11	2.51	–
CL3108.Contig2	bZIP transcription factor	Setaria italica	2.20E-33	2.66	–
CL12321.Contig3	Basic leucine zipper	Arabidopsis thaliana	5.10E-10	2.74	–
CL7470.Contig1	STRUBBELIG family receptor protein kinase	Zea mays	2.70E-180	2.81	–
CL8093.Contig1	Calmodulin-binding protein	Arabidopsis thaliana	4.60E-83	2.82	–
Unigene22524	calcium-binding protein CML30	Arabidopsis thaliana	1.80E-11	2.92	2.69
CL4719.Contig2	Respiratory burst oxidase homolog protein E	Arabidopsis thaliana	2.80E-281	2.98	–
Unigene20102	polcalcin Jun o 2	Zea mays	1.30E-07	3.02	2.45
Unigene22489	LRR receptor-like serine/threonine-protein kinase	Setaria italica	1.20E-234	3.05	–
Unigene22004	Protein STRUBBELIG-RECEPTOR FAMILY 7	Arabidopsis thaliana	6.90E-43	3.41	–
Unigene34056	receptor protein kinase ZmPK1	Zea mays	6.80E-38	3.42	–
CL1412.Contig1	U-box domain-containing protein	Arabidopsis thaliana	1.10E-72	3.68	–
CL1672.Contig1	serine/threonine-protein kinase	Setaria italica	3.40E-33	3.84	–
Unigene17371	transcription factor BHLH148	Sorghum bicolor	0.00E+00	3.93	–
Unigene15066	Transcription factor ABORTED MICROSPORES	Arabidopsis thaliana	7.70E-18	3.97	–
CL4655.Contig1	receptor-like protein kinase	Zea mays	3.46E-128	4.31	–

Table S1 (Continued)

Gene ID	Description	Blast species	e_value	WS-9_FC	RW_FC
CL3365.Contig3	Protein STRUBBELIG-RECEPTOR FAMILY	Arabidopsis thaliana	4.20E-40	4.44	–
CL12929.Contig1	Protein TIFY 11c	Oryza sativa	6.60E-40	4.69	–
Unigene17931	pathogenesis-related protein	Setaria italica	3.20E-18	4.71	–
Unigene1014	pathogenesis-related protein 1	Zea mays	1.10E-13	4.80	–
CL5034.Contig1	pathogenesis-related protein	Zea mays	3.40E-42	6.09	3.87
CL11032.Contig1	L-type lectin-domain containing receptor kinase	Brachypodium distachyo	7.80E-77	7.03	–
Unigene41884	disease resistance RPP13-like protein	Zea mays	4.60E-45	7.97	–
Unigene34151	ZIM transcription factor	Zea mays	8.00E-35	8.12	–
Unigene59500	LRR receptor-like serine/threonine-protein kinase	Arabidopsis thaliana	2.50E-13	8.34	–
Unigene10648	disease resistance protein RPM1-like	Zea mays	3.00E-53	8.68	–
Unigene58833	Disease resistance protein RPS2	Arabidopsis thaliana	3.00E-11	8.75	7.88
Unigene17932	Pathogenesis-related protein PRMS	Zea mays	4.70E-32	9.87	–
Unigene20764	L-type lectin-domain containing receptor kinase	Arabidopsis thaliana	1.10E-138	10.25	–
Unigene60151	LRR receptor-like serine/threonine-protein kinase	Aegilops tauschii	5.20E-16	–	7.99
Unigene54434	NBS-LRR type protein	Oryza sativa	7.60E-10	–	7.93
Unigene34533	LRR receptor-like serine/threonine-protein kinase	Zea mays	5.20E-16	–	2.78
Unigene13522	G-type lectin S-receptor-like serine/threonine-protein kinase RLK1	Arabidopsis thaliana	5.70E-33	–	-8.37
CL5366.Contig1	LRR receptor-like serine/threonine-protein kinase	Sorghum bicolor	7.00E-22	–	7.98
**Phenylpropanoid biosynthesis**					
Unigene37048	4-coumarate-CoA ligase	Zea mays	3.60E-09	-9.09	–
Unigene21145	O-methyltransferase ZRP4	Zea mays	1.20E-81	-5.05	–
CL10556.Contig3	cytochrome P450 CYP78A52	Zea mays	2.90E-30	-3.63	-3.68
Unigene33285	cytokinin-O-glucosyltransferase	Zea mays	1.40E-33	-3.60	–
CL10556.Contig2	cytochrome P450 superfamily protein	Zea mays	3.00E-83	-3.45	-3.17
Unigene21058	RNA-binding protein	Sorghum bicolor	2.00E-71	-3.33	–
CL10639.Contig5	beta-glucosidase	Sorghum bicolor	6.00E-51	-3.05	–
CL9816.Contig1	Shikimate O-hydroxycinnamoyltransferase	Nicotiana tabacum	2.00E-51	-2.67	–
Unigene27838	Beta-glucosidase 45	Arabidopsis thaliana	1.30E-23	-2.36	–
Unigene663	ferulate 5-hydroxylase	Miscanthus x giganteus	2.80E-241	2.36	–
Unigene28815	Serine carboxypeptidase-like 18	Arabidopsis thaliana	2.00E-56	2.43	–
Unigene27759	cinnamyl alcohol dehydrogenase 5	Oryza sativa	3.60E-61	2.50	–
Unigene28830	Serine carboxypeptidase-like 18	Arabidopsis thaliana	1.20E-116	3.16	–
CL10140.Contig2	beta-glucosidase	Setaria italica	0.00E+00	3.38	–
CL2409.Contig2	Phenylalanine ammonia-lyase	Oryza sativa	0.00E+00	3.48	–
Unigene18463	phenylalanine ammonia lyase	Saccharum officinarum	2.50E-70	3.59	–
Unigene16984	Peroxidase 12	Arabidopsis thaliana	3.00E-19	3.75	–
CL10335.Contig1	cinnamoyl-CoA reductase-like protein	Miscanthus x giganteus	7.80E-145	3.80	–
CL10269.Contig4	Trans-cinnamate 4-monooxygenase	Glycine max	9.30E-224	3.95	–
CL11864.Contig1	cinnamoyl-CoA reductase-like protein	Miscanthus x giganteus	4.30E-71	4.07	–
Unigene25289	3'-N-debenzoyl-2'-deoxytaxol N-benzoyltransferase	Taxus canadensis	3.80E-17	4.24	–
CL2285.Contig8	exhydrolase II	Zea mays	3.00E-284	4.29	–
Unigene19824	Peroxidase	Zea mays	3.80E-56	5.22	–
Unigene23772	Shikimate O-hydroxycinnamoyltransferase	Nicotiana tabacum	1.90E-48	5.28	–
CL7850.Contig1	Beta-glucosidase	Oryza sativa	7.70E-193	5.49	–
Unigene18654	cinnamate 4-hydroxylase	Miscanthus x giganteus	7.30E-15	5.51	–
Unigene13757	agmatine coumaroyltransferase-1-like	Zea mays	3.70E-42	5.71	–
CL10269.Contig2	Trans-cinnamate 4-monooxygenase	Glycine max	3.90E-19	5.74	–
Unigene18590	Peroxidase	Triticum aestivum	1.60E-10	5.80	–
Unigene16778	Shikimate O-hydroxycinnamoyltransferase	Arabidopsis thaliana	6.70E-63	6.75	–
Unigene27760	mannitol dehydrogenase	Zea mays	1.80E-163	7.61	–
Unigene40037	Peroxidase	Arabidopsis thaliana	4.30E-29	8.02	–
Unigene52365	Peroxidase	Arabidopsis thaliana	1.10E-40	8.03	–

Table S1 (Continued)

Gene ID	Description	Blast species	e_value	WS-9_FC	RW_FC
Unigene45671	agmatine coumaroyltransferase	Zea mays	4.50E-31	8.84	–
Unigene19825	Peroxidase	Zea mays	2.20E-08	8.87	–
Unigene25358	Peroxidase N	Armoracia rusticana	9.30E-19	9.38	–
**Endocytosis**					
CL11154.Contig1	Thaumatin-like protein	Pyrus pyrifolia	4.90E-64	-5.59	–
CL11154.Contig2	Thaumatin-like protein	Pyrus pyrifolia	4.90E-64	-3.82	–
CL5236.Contig2	protein targeted either to mitochondria or chloroplast proteins	Sorghum bicolor	9.40E-99	-3.17	–
CL1882.Contig1	myosin-9	Sorghum bicolor	0.00E+00	-2.50	–
Unigene20869	36.4 kDa proline-rich protein	Zea mays	3.80E-37	-2.37	2.44
CL2442.Contig1	swi5-dependent recombination DNA repair protein	Setaria italica	6.00E-151	2.30	–
CL12818.Contig3	Heat shock cognate 70 kDa protein	Petunia hybrida	1.00E-40	2.44	–
Unigene28547	heat shock 70 kDa protein 1	Zea mays	0.00E+00	2.56	–
CL12818.Contig2	Heat shock cognate 70 kDa protein	Petunia hybrida	4.30E-46	2.69	–
Unigene12711	mediator of RNA polymerase II transcription subunit	Oryza brachyantha	1.50E-08	2.75	–
CL12818.Contig5	Heat shock cognate 70 kDa protein	Petunia hybrida	9.30E-98	2.85	–
CL5161.Contig1	Non-specific lipid-transfer protein	Zea mays	6.10E-35	2.96	–
CL12818.Contig4	Heat shock cognate 70 kDa protein	Petunia hybrida	6.10E-82	3.00	–
Unigene26939	E3 ubiquitin ligase BIG BROTHER-related	Sorghum bicolor	8.00E-70	3.11	–
Unigene21575	Heat shock 70 kDa protein	Zea mays	4.00E-22	3.12	–
Unigene21574	heat shock protein	Zea mays	1.11E-77	3.17	–
Unigene19459	Heat shock cognate 70 kDa protein	Petunia hybrida	2.20E-11	3.18	–
Unigene19457	Heat shock 70 kDa protein	Chlamydomonas reinharc	1.00E-14	3.42	–
Unigene10070	extensin	Zea mays	5.00E-59	3.46	–
Unigene14468	mediator of RNA polymerase II transcription subunit	Arabidopsis thaliana	5.50E-12	3.64	–
CL9329.Contig1	BAG family molecular chaperone regulator 6	Zea mays	0.00E+00	3.76	–
Unigene191	F-box/kelch-repeat protein	Setaria italica	0.00E+00	4.28	–
Unigene21219	Blue copper protein	Pisum sativum	1.60E-21	4.38	–
Unigene19458	mediator of RNA polymerase II transcription subunit	Arabidopsis thaliana	9.00E-19	4.43	–
Unigene24419	Amino acid permease 8	Arabidopsis thaliana	3.60E-20	5.27	–
Unigene17519	pollen-specific leucine-rich repeat extensin-like protein	Setaria italica	2.00E-95	5.80	–
Unigene17860	vegetative cell wall protein gp1	Setaria italica	4.50E-14	7.10	–
CL6142.Contig2	dynamin-related protein 5A	Sorghum bicolor	2.00E-150	7.99	–
Unigene36989	carboxylesterase	Setaria italica	1.30E-44	9.09	–
Unigene22187	30S ribosomal protein	Sorghum bicolor	5.00E-18	–	2.17
**Spliceosome**					
Unigene21058	RNA-binding protein	Sorghum bicolor	2.00E-71	-3.33	–
Unigene2660	DUF1645 family protein	Zea mays	1.00E-27	-2.52	–
Unigene29886	pleiotropic drug resistance protein	Zea mays	0.00E+00	2.12	–
CL335.Contig3	resistance gene analogue	Saccharum officinarum	9.00E-174	2.20	–
CL11369.Contig1	dehydrin HIRD11	Sorghum bicolor	0.00E+00	2.23	–
Unigene29887	ABC transporter G family member 36	Oryza sativa	0.00E+00	2.31	–
CL12818.Contig3	Heat shock cognate 70 kDa protein	Petunia hybrida	1.00E-40	2.44	–
Unigene28547	heat shock 70 kDa protein 1	Zea mays	0.00E+00	2.56	–
CL12818.Contig2	Heat shock cognate 70 kDa protein	Petunia hybrida	4.30E-46	2.69	–
CL629.Contig1	ABC transporter G family member	Oryza sativa	0.00E+00	2.70	–
Unigene12711	mediator of RNA polymerase II transcription subunit	Oryza brachyantha	1.50E-08	2.75	–
CL12818.Contig5	Heat shock cognate 70 kDa protein	Petunia hybrida	9.30E-98	2.85	–
CL12818.Contig4	Heat shock cognate 70 kDa protein	Petunia hybrida	6.10E-82	3.00	–
Unigene19686	loricrin	Saccharum officinarum	6.90E-17	3.05	–
Unigene21575	Heat shock 70 kDa protein	Zea mays	4.00E-22	3.12	–
Unigene33310	keratin-associated protein	Sorghum bicolor	0.00E+00	3.13	–
Unigene21574	heat shock protein	Zea mays	1.11E-77	3.17	–

Table S1 (Continued)

Gene ID	Description	Blast species	e_value	WS-9_FC	RW_FC
Unigene19459	Heat shock cognate 70 kDa protein	Petunia hybrida	2.20E-11	3.18	–
CL629.Contig2	ABC transporter G family member	Oryza sativa	1.90E-85	3.19	–
Unigene19457	Heat shock 70 kDa protein	Chlamydomonas reinharc	1.00E-14	3.42	–
CL2470.Contig1	glucan endo-1,3-beta-glucosidase	Zea mays	3.64E-171	3.47	–
Unigene14468	mediator of RNA polymerase II transcription subunit	Arabidopsis thaliana	5.50E-12	3.64	–
Unigene17998	hepatoma-derived growth factor-related protein	Sorghum bicolor	0.00E+00	4.31	–
Unigene19458	mediator of RNA polymerase II transcription subunit	Arabidopsis thaliana	9.00E-19	4.43	–
CL11007.Contig1	ABC transporter G family member	Triticum urartu	4.50E-205	5.90	–
Unigene63788	ABC transporter G family member	Oryza sativa	1.40E-27	7.97	–
Unigene6985	ABC transporter G family member	Oryza sativa	1.60E-45	8.10	–
Unigene15413	DUF679 domain membrane protein	Zea mays	4.00E-103	8.72	–
CL5005.Contig1	expansin-B3-like	Zea mays	2.00E-135	–	3.55
**Glycerophospholipid metabolism**					
CL11154.Contig1	Thaumatin-like protein	Pyrus pyrifolia	4.90E-64	-5.59	–
CL11154.Contig2	Thaumatin-like protein	Pyrus pyrifolia	4.90E-64	-3.82	–
CL8930.Contig2	glycerophosphodiester phosphodiesterase GDPD6	Oryza brachyantha	2.60E-30	-8.87	–
CL5236.Contig2	protein targeted either to mitochondria or chloroplast proteins	Sorghum bicolor	9.40E-99	-3.17	–
CL1882.Contig1	myosin-9	Sorghum bicolor	0.00E+00	-2.50	–
Unigene20869	36.4 kDa proline-rich protein	Zea mays	3.80E-37	-2.37	2.44
CL2442.Contig1	swi5-dependent recombination DNA repair protein	Setaria italica	6.00E-151	2.30	–
CL9247.Contig13	phosphoethanolamine N-methyltransferase	Oryza sativa	3.00E-260	2.40	–
Unigene19383	Glycerol-3-phosphate 2-O-acyltransferase	Arabidopsis thaliana	3.10E-107	2.58	–
CL5161.Contig1	Non-specific lipid-transfer protein	Zea mays	6.10E-35	2.96	–
Unigene26939	E3 ubiquitin ligase BIG BROTHER-related	Sorghum bicolor	8.00E-70	3.11	–
Unigene19384	glycerol-3-phosphate 2-O-acyltransferase	Setaria italica	1.90E-110	3.19	–
Unigene10070	extensin	Zea mays	5.00E-59	3.46	–
CL9329.Contig1	BAG family molecular chaperone regulator 6	Zea mays	0.00E+00	3.76	–
Unigene191	F-box/kelch-repeat protein	Setaria italica	0.00E+00	4.28	–
Unigene21219	Blue copper protein	Pisum sativum	1.60E-21	4.38	–
Unigene18840	Glycerol-3-phosphate acyltransferase	Arabidopsis thaliana	8.70E-37	4.55	–
Unigene24419	Amino acid permease 8	Arabidopsis thaliana	3.60E-20	5.27	–
Unigene17519	pollen-specific leucine-rich repeat extensin-like protein	Setaria italica	2.00E-95	5.80	–
Unigene17860	vegetative cell wall protein gp1	Setaria italica	4.50E-14	7.10	–
Unigene38762	phosphoesterase	Saccharum officinarum	7.00E-20	9.07	–
Unigene36989	carboxylesterase	Setaria italica	1.30E-44	9.09	–
Unigene6969	lecithin-cholesterol acyltransferase	Setaria italica	2.00E-12	–	-7.88
Unigene22187	30S ribosomal protein	Sorghum bicolor	5.00E-18	–	2.17
**Stilbenoid, diarylheptanoid and gingerol biosynthesis**					
Unigene21145	O-methyltransferase ZRP4	Zea mays	1.20E-81	-5.05	–
CL10556.Contig1	Cytochrome P450	Arabidopsis thaliana	5.50E-160	-4.21	–
CL10556.Contig3	cytochrome P450 CYP78A52	Zea mays	2.90E-30	-3.63	-3.68
CL10556.Contig2	cytochrome P450 superfamily protein	Zea mays	3.00E-83	-3.45	-3.17
Unigene23660	cytochrome P450 superfamily protein	Zea mays	1.10E-21	-3.40	-3.41
Unigene23658	Cytochrome P450	Arabidopsis thaliana	1.50E-32	-3.39	-2.60
Unigene23659	cytochrome P450 CYP78A54	Zea mays	3.50E-42	-3.33	-2.43
CL9816.Contig1	Shikimate O-hydroxycinnamoyltransferase	Nicotiana tabacum	2.00E-51	-2.67	–
CL13117.Contig1	O-methyltransferase-like protein	Saccharum officinarum	2.00E-198	-2.38	–
Unigene26449	Isoflavone 2'-hydroxylase	Medicago truncatula	1.80E-64	3.01	–
Unigene33150	indole-2-monooxygenase	Zea mays	1.10E-14	3.19	–
CL10269.Contig4	Trans-cinnamate 4-monooxygenase	Glycine max	9.30E-224	3.95	–
Unigene25289	3'-N-debenzoyl-2'-deoxytaxol N-benzoyltransferase	Taxus canadensis	3.80E-17	4.24	–
Unigene18706	Cytochrome P450	Persea americana	2.40E-109	4.59	–

Table S1 (Continued)

Gene ID	Description	Blast species	e_value	WS-9_FC	RW_FC
Unigene23772	Shikimate O-hydroxycinnamoyltransferase	Nicotiana tabacum	1.90E-48	5.28	–
Unigene18654	cinnamate 4-hydroxylase	Miscanthus x giganteus	7.30E-15	5.51	–
Unigene13757	agmatine coumaroyltransferase-1-like	Zea mays	3.70E-42	5.71	–
Unigene17341	indole-2-monooxygenase	Zea mays	1.70E-26	5.74	–
CL10269.Contig2	Trans-cinnamate 4-monooxygenase	Glycine max	3.90E-19	5.74	–
Unigene16778	Shikimate O-hydroxycinnamoyltransferase	Arabidopsis thaliana	6.70E-63	6.75	–
Unigene38259	cytochrome P450	Saccharum officinarum	1.90E-61	8.79	–
Unigene45671	agmatine coumaroyltransferase	Zea mays	4.50E-31	8.84	–
Unigene16462	5-pentadecatrienyl resorcinol O-methyltransferase	Sorghum bicolor	5.90E-109	–	3.07
**Ether lipid metabolism**					
CL11154.Contig1	Thaumatin-like protein	Pyrus pyrifolia	4.90E-64	-5.59	–
CL11154.Contig2	Thaumatin-like protein	Pyrus pyrifolia	4.90E-64	-3.82	–
CL5236.Contig2	protein targeted either to mitochondria or chloroplast proteins	Sorghum bicolor	9.40E-99	-3.17	–
CL1882.Contig1	myosin-9	Sorghum bicolor	0.00E+00	-2.50	–
Unigene20869	36.4 kDa proline-rich protein	Zea mays	3.80E-37	-2.37	2.44
CL2442.Contig1	swi5-dependent recombination DNA repair protein	Setaria italica	6.00E-151	2.30	–
CL5161.Contig1	Non-specific lipid-transfer protein	Zea mays	6.10E-35	2.96	–
Unigene26939	E3 ubiquitin ligase BIG BROTHER-related	Sorghum bicolor	8.00E-70	3.11	–
Unigene10070	extensin	Zea mays	5.00E-59	3.46	–
CL9329.Contig1	BAG family molecular chaperone regulator 6	Zea mays	0.00E+00	3.76	–
Unigene191	F-box/kelch-repeat protein	Setaria italica	0.00E+00	4.28	–
Unigene21219	Blue copper protein	Pisum sativum	1.60E-21	4.38	–
Unigene24419	Amino acid permease 8	Arabidopsis thaliana	3.60E-20	5.27	–
Unigene17519	pollen-specific leucine-rich repeat extensin-like protein	Setaria italica	2.00E-95	5.80	–
Unigene17860	vegetative cell wall protein gp1	Setaria italica	4.50E-14	7.10	–
Unigene38762	phosphoesterase	Saccharum officinarum	7.00E-20	9.07	–
Unigene36989	carboxylesterase	Setaria italica	1.30E-44	9.09	–
Unigene22187	30S ribosomal protein	Sorghum bicolor	5.00E-18	–	2.17
**Starch and sucrose metabolism**					
CL10140.Contig2	beta-glucosidase	Setaria italica	0.00E+00	3.38	–
CL11154.Contig1	Thaumatin-like protein	Pyrus pyrifolia	4.90E-64	-5.59	–
CL11154.Contig2	Thaumatin-like protein	Pyrus pyrifolia	4.90E-64	-3.82	–
CL10639.Contig5	beta-glucosidase	Sorghum bicolor	6.00E-51	-3.05	–
Unigene27838	Beta-glucosidase 45	Arabidopsis thaliana	1.30E-23	-2.36	–
CL131.Contig3	soluble acid invertase	Saccharum officinarum	0.00E+00	2.12	–
Unigene22946	Beta-amylase 1	Arabidopsis thaliana	7.60E-10	2.66	–
CL4049.Contig1	galacturonosyltransferase-like	Arabidopsis thaliana	2.50E-117	2.71	–
Unigene27924	beta-amylase	Zea mays	0.00E+00	3.08	–
Unigene26939	E3 ubiquitin ligase BIG BROTHER-related	Sorghum bicolor	8.00E-70	3.11	–
CL9767.Contig2	beta-amylase	Saccharum officinarum	2.90E-205	3.21	–
Unigene2276	trehalose-phosphate phosphatase 9	Oryza sativa	5.90E-17	3.69	–
Unigene23525	trehalose-phosphate phosphatase 4	Oryza sativa	1.70E-33	4.18	–
Unigene191	F-box/kelch-repeat protein	Setaria italica	0.00E+00	4.28	–
CL2285.Contig8	exhydrolase II	Zea mays	3.00E-284	4.29	–
CL7850.Contig1	Beta-glucosidase	Oryza sativa	7.70E-193	5.49	–
Unigene23524	trehalose-phosphate phosphatase 9	Oryza sativa	3.10E-46	5.51	–
Unigene17860	vegetative cell wall protein gp1	Setaria italica	4.50E-14	7.10	–
Unigene35539	cellulase (Cel24A)	Zea mays	1.62E-107	7.95	–
Unigene47247	Endoglucanase 5	Oryza sativa	3.40E-15	8.57	–
**Alanine, aspartate and glutamate metabolism**					
Unigene24406	aldehyde dehydrogenase	Saccharum officinarum	1.60E-106	2.06	–
CL11737.Contig2	alanine aminotransferase	Zea mays	3.30E-249	2.21	–

Table S1 (Continued)

Gene ID	Description	Blast species	e_value	WS-9_FC	RW_FC
CL6765.Contig1	glutamate decarboxylase	Setaria italica	1.30E-248	2.27	–
Unigene23719	glutamate dehydrogenase	Oryza sativa	6.50E-220	2.42	–
Unigene3245	aldehyde dehydrogenase	Cleistogenes songorica	1.00E-302	3.27	–
CL5141.Contig1	glutamine synthetase	Saccharum officinarum	4.20E-23	3.41	–
Unigene19529	asparagine synthetase	Zea mays	0.00E+00	3.76	–
Unigene19530	asparagine synthetase	Zea mays	0.00E+00	3.84	–
Unigene19652	glutamine synthetase	Saccharum officinarum	5.43E-161	5.03	–
Unigene19651	glutamine synthetase cytosolic isozyme	Setaria italica	1.70E-08	5.99	–
Unigene1326	asparagine synthetase	Zea mays	0.00E+00	–	-2.58
Unigene14845	Glutamate synthase 2 [NADH]	Oryza sativa	6.70E-103	–	2.59
Unigene14471	Glutamate synthase 1 [NADH]	Oryza sativa	6.10E-95	–	2.97
Unigene35100	Glutamate synthase 1 [NADH]	Oryza sativa	6.20E-64	–	3.25
Unigene42648	Glutamate synthase 2 [NADH]	Oryza sativa	8.70E-16	–	3.26
Unigene52905	glutamate synthase (NADPH/NADH)	Sorghum bicolor	2.76E-92	–	3.37
Unigene14844	Glutamate synthase 2 [NADH]	Oryza sativa	1.50E-98	–	3.44
Unigene61922	Glutamate synthase 1 [NADH]	Arabidopsis thaliana	1.40E-44	–	3.49
Unigene41566	Glutamate synthase 2 [NADH]	Oryza sativa	1.60E-22	–	3.68
**Protein processing in endoplasmic reticulum**					
Unigene21058	RNA-binding protein	Sorghum bicolor	2.00E-71	-3.33	–
CL12818.Contig3	Heat shock cognate 70 kDa protein	Petunia hybrida	1.00E-40	2.44	–
Unigene28547	heat shock 70 kDa protein 1	Zea mays	0.00E+00	2.56	–
CL12818.Contig2	Heat shock cognate 70 kDa protein	Petunia hybrida	4.30E-46	2.69	–
Unigene12711	mediator of RNA polymerase II transcription subunit	Oryza brachyantha	1.50E-08	2.75	–
CL12818.Contig5	Heat shock cognate 70 kDa protein	Petunia hybrida	9.30E-98	2.85	–
CL12818.Contig4	Heat shock cognate 70 kDa protein	Petunia hybrida	6.10E-82	3.00	–
Unigene34139	18.0 kDa class II heat shock protein	Oryza sativa	2.00E-13	3.07	–
Unigene21575	Heat shock 70 kDa protein	Zea mays	4.00E-22	3.12	–
Unigene21574	heat shock protein	Zea mays	1.11E-77	3.17	–
Unigene19459	Heat shock cognate 70 kDa protein	Petunia hybrida	2.20E-11	3.18	–
Unigene19457	Heat shock 70 kDa protein	Chlamydomonas reinhar	1.00E-14	3.42	–
Unigene14468	mediator of RNA polymerase II transcription subunit	Arabidopsis thaliana	5.50E-12	3.64	–
Unigene19458	mediator of RNA polymerase II transcription subunit	Arabidopsis thaliana	9.00E-19	4.43	–
Unigene4747	U-box domain-containing protein	Arabidopsis thaliana	1.10E-19	8.02	–
Unigene46216	Cysteine-rich receptor-like protein kinase	Aegilops tauschii	4.10E-16	8.16	–
Unigene30497	sulfate transporter 3.4	Arabidopsis thaliana	2.30E-21	9.27	–
**Nitrogen metabolism**					
Unigene23719	glutamate dehydrogenase	Oryza sativa	6.50E-220	2.42	–
CL6643.Contig1	Electron transfer flavoprotein subunit alpha	Oryza sativa	1.20E-145	2.43	–
CL5141.Contig1	glutamine synthetase	Saccharum officinarum	4.20E-23	3.41	–
Unigene19529	asparagine synthetase	Zea mays	0.00E+00	3.76	–
Unigene19530	asparagine synthetase	Zea mays	0.00E+00	3.84	–
Unigene19652	glutamine synthetase	Saccharum officinarum	5.43E-161	5.03	–
Unigene19651	glutamine synthetase cytosolic isozyme	Setaria italica	1.70E-08	5.99	–
Unigene1326	asparagine synthetase	Zea mays	0.00E+00	–	-2.58
Unigene14845	Glutamate synthase 2 [NADH]	Oryza sativa	6.70E-103	–	2.59
Unigene14471	Glutamate synthase 1 [NADH]	Oryza sativa	6.10E-95	–	2.97
Unigene35100	Glutamate synthase 1 [NADH]	Oryza sativa	6.20E-64	–	3.25
Unigene42648	Glutamate synthase 2 [NADH]	Oryza sativa	8.70E-16	–	3.26
Unigene52905	glutamate synthase (NADPH/NADH)	Sorghum bicolor	2.76E-92	–	3.37
Unigene14844	Glutamate synthase 2 [NADH]	Oryza sativa	1.50E-98	–	3.44
Unigene61922	Glutamate synthase 1 [NADH]	Arabidopsis thaliana	1.40E-44	–	3.49
Unigene41566	Glutamate synthase 2 [NADH]	Oryza sativa	1.60E-22	–	3.68

Table S1 (Continued)

Gene ID	Description	Blast species	e_value	WS-9_FC	RW_FC
**ABC transporters**					
Unigene29886	pleiotropic drug resistance protein	Zea mays	0.00E+00	2.12	–
Unigene29887	ABC transporter G family member 36	Oryza sativa	0.00E+00	2.31	–
Unigene17650	Cysteine-rich receptor-like protein kinase	Arabidopsis thaliana	1.90E-11	2.51	–
CL629.Contig1	ABC transporter G family member	Oryza sativa	0.00E+00	2.70	–
CL629.Contig2	ABC transporter G family member	Oryza sativa	1.90E-85	3.19	–
Unigene20986	serine/threonine-protein kinase	Setaria italica	3.00E-69	3.40	–
CL1412.Contig1	U-box domain-containing protein	Arabidopsis thaliana	1.10E-72	3.68	–
CL1672.Contig1	serine/threonine-protein kinase	Setaria italica	3.40E-33	3.84	–
Unigene13019	ABC transporter A family member	Brachypodium distachyo	3.40E-169	4.27	–
Unigene7599	ABC transporter B family member	Zea mays	1.10E-104	4.59	–
Unigene6331	multidrug resistance protein	Zea mays	3.20E-40	5.77	–
CL11007.Contig1	ABC transporter G family member	Triticum urartu	4.50E-205	5.90	–
CL5721.Contig1	ABC transporter B family member	Arabidopsis thaliana	9.10E-10	8.80	–
CL328.Contig3	multidrug resistance protein	Sorghum bicolor	0.00E+00	8.98	–
Unigene13522	G-type lectin S-receptor-like serine/threonine-protein kinase RLK1	Arabidopsis thaliana	5.70E-33	–	-8.37
**Flavonoid biosynthesis**					
CL10556.Contig1	Cytochrome P450	Arabidopsis thaliana	5.50E-160	-4.21	–
CL10556.Contig3	cytochrome P450 CYP78A52	Zea mays	2.90E-30	-3.63	–
CL10556.Contig2	cytochrome P450 superfamily protein	Zea mays	3.00E-83	-3.45	–
Unigene23658	Cytochrome P450	Arabidopsis thaliana	1.50E-32	-3.39	–
CL9816.Contig1	Shikimate O-hydroxycinnamoyltransferase	Nicotiana tabacum	2.00E-51	-2.67	–
CL10269.Contig4	Trans-cinnamate 4-monooxygenase	Glycine max	9.30E-224	3.95	–
Unigene25289	3'-N-debenzoyl-2'-deoxytaxol N-benzoyltransferase	Taxus canadensis	3.80E-17	4.24	–
Unigene23772	Shikimate O-hydroxycinnamoyltransferase	Nicotiana tabacum	1.90E-48	5.28	–
Unigene18654	cinnamate 4-hydroxylase	Miscanthus x giganteus	7.30E-15	5.51	–
Unigene13757	agmatine coumaroyltransferase-1-like	Zea mays	3.70E-42	5.71	–
CL10269.Contig2	Trans-cinnamate 4-monooxygenase	Glycine max	3.90E-19	5.74	–
Unigene23154	indole-2-monooxygenase	Zea mays	1.40E-90	6.20	–
Unigene16778	Shikimate O-hydroxycinnamoyltransferase	Arabidopsis thaliana	6.70E-63	6.75	–
Unigene45671	agmatine coumaroyltransferase	Zea mays	4.50E-31	8.84	–
CL4264.Contig4	chalcone synthase	Sorghum bicolor	1.00E-88	9.81	–
**Phenylalanine metabolism**					
Unigene37048	4-coumarate-CoA ligase	Zea mays	3.60E-09	-9.09	–
Unigene21058	RNA-binding protein	Sorghum bicolor	2.00E-71	-3.33	–
CL2409.Contig2	Phenylalanine ammonia-lyase	Oryza sativa	0.00E+00	3.48	–
Unigene18463	phenylalanine ammonia lyase	Saccharum officinarum	2.50E-70	3.59	–
Unigene16984	Peroxidase 12	Arabidopsis thaliana	3.00E-19	3.75	–
CL10269.Contig4	Trans-cinnamate 4-monooxygenase	Glycine max	9.30E-224	3.95	–
Unigene19824	Peroxidase	Zea mays	3.80E-56	5.22	–
Unigene18654	cinnamate 4-hydroxylase	Miscanthus x giganteus	7.30E-15	5.51	–
CL10269.Contig2	Trans-cinnamate 4-monooxygenase	Glycine max	3.90E-19	5.74	–
Unigene18590	Peroxidase	Triticum aestivum	1.60E-10	5.80	–
Unigene40037	Peroxidase	Arabidopsis thaliana	4.30E-29	8.02	–
Unigene52365	Peroxidase	Arabidopsis thaliana	1.10E-40	8.03	–
Unigene19825	Peroxidase	Zea mays	2.20E-08	8.87	–
Unigene25358	Peroxidase N	Armoracia rusticana	9.30E-19	9.38	–
**Pyrimidine metabolism**					
CL11925.Contig2	NAC domain-containing protein	Oryza sativa	1.90E-55	7.10	–
Unigene25178	Beta-ureidopropionase	Triticum urartu	2.50E-216	2.08	–
Unigene25808	aspartic proteinase nepenthesin	Setaria italica	1.70E-94	2.31	–
Unigene2767	nuclear factor YB2	Sorghum bicolor	2.50E-80	2.36	–

Table S1 (Continued)

Gene ID	Description	Blast species	e_value	WS-9_FC	RW_FC
Unigene24235	Disease resistance protein RGA2	Solanum bulbocastanum	2.10E-08	2.58	–
CL4351.Contig1	Disease resistance protein RGA2	Solanum bulbocastanum	6.30E-10	2.67	–
Unigene17751	CTP synthase	Zea mays	2.00E-101	2.77	–
CL317.Contig1	CTP synthase 1-like isoform	Durio zibethinus	3.00E-38	2.99	–
Unigene2239	CTP synthase	Zea mays	0.00E+00	3.30	–
Unigene1583	galactinol synthase	Setaria italica	1.00E-65	3.37	–
CL13350.Contig1	CHP-rich zinc finger protein-like	Oryza sativa	4.00E-35	3.72	–
Unigene18699	disease resistance protein RGA4	Aegilops tauschii	9E-15	6.91	–
Unigene54434	NBS-LRR type protein	Oryza sativa	7.60E-10	–	7.93
**Limonene and pinene degradation**					
CL10556.Contig1	Cytochrome P450	Arabidopsis thaliana	5.50E-160	-4.21	–
CL10556.Contig3	cytochrome P450 CYP78A52	Zea mays	2.90E-30	-3.63	-3.68
CL10556.Contig2	cytochrome P450 superfamily protein	Zea mays	3.00E-83	-3.45	-3.17
Unigene23660	cytochrome P450 superfamily protein	Zea mays	1.10E-21	-3.40	-3.41
Unigene23658	Cytochrome P450	Arabidopsis thaliana	1.50E-32	-3.39	-2.60
Unigene23659	cytochrome P450 CYP78A54	Zea mays	3.50E-42	-3.33	-2.43
Unigene26449	Isoflavone 2'-hydroxylase	Medicago truncatula	1.80E-64	3.01	–
Unigene33150	indole-2-monooxygenase	Zea mays	1.10E-14	3.19	–
Unigene18706	Cytochrome P450	Persea americana	2.40E-109	4.59	–
Unigene17341	indole-2-monooxygenase	Zea mays	1.70E-26	5.74	–
Unigene38259	cytochrome P450	Saccharum officinarum	1.90E-61	8.79	–
**Amino sugar and nucleotide sugar metabolism**					
CL4049.Contig1	galacturonosyltransferase-like	Arabidopsis thaliana	2.50E-117	2.71	–
CL6067.Contig1	DUF1677 domain-containing protein	Cephalotus follicularis	5.00E-40	-2.94	–
Unigene22255	Chitinase 2	Oryza sativa	7.90E-70	3.67	–
CL13120.Contig2	chitinase	Saccharum officinarum	1.20E-154	3.86	–
Unigene343	Xylanase inhibitor protein	Triticum aestivum	2.50E-94	6.60	–
Unigene19626	Alpha-L-arabinofuranosidase	Arabidopsis thaliana	8.90E-14	8.08	–
Unigene26173	Xylanase inhibitor protein 1	Oryza sativa	1.40E-88	8.10	–
Unigene10626	chitinase B	Saccharum officinarum	9.50E-43	8.75	–
Unigene30497	sulfate transporter 3.4	Arabidopsis thaliana	2.30E-21	9.27	–
Unigene19350	Xylanase inhibitor protein	Triticum aestivum	2.00E-26	12.23	–
**Isoflavonoid biosynthesis**					
Unigene24078	Anthocyanidin 5,3-O-glucosyltransferase	Rosa hybrid	3.20E-38	2.28	–
Unigene24079	Anthocyanidin 5,3-O-glucosyltransferase	Rosa hybrid	5.80E-56	2.58	–
Unigene23154	indole-2-monooxygenase	Zea mays	1.40E-90	6.20	–
Unigene3564	gibberellin receptor GID1L2	Zea mays	0.00E+00	6.25	–
Unigene36989	carboxylesterase	Setaria italica	1.30E-44	9.09	–
CL10556.Contig3	cytochrome P450 CYP78A52	Zea mays	2.90E-30	–	-3.68
CL10556.Contig2	cytochrome P450 superfamily protein	Zea mays	3.00E-83	–	-3.17
Unigene23658	Cytochrome P450	Arabidopsis thaliana	1.50E-32	–	-2.60
CL4209.Contig1	gibberellin 20 oxidase	Zea mays	3.70E-187	–	2.41
CL4264.Contig4	chalcone synthase	Sorghum bicolor	1.00E-88	–	9.63
**Purine metabolism**					
Unigene25808	aspartic proteinase nepenthesin	Setaria italica	1.70E-94	2.31	–
Unigene2767	nuclear factor YB2	Sorghum bicolor	2.50E-80	2.36	–
CL8.Contig1	Inosine-5'-monophosphate dehydrogenase	Vigna unguiculata	9.70E-174	2.51	–
Unigene24235	Disease resistance protein RGA2	Solanum bulbocastanum	2.10E-08	2.58	–
CL4351.Contig1	Disease resistance protein RGA2	Solanum bulbocastanum	6.30E-10	2.67	–
Unigene1583	galactinol synthase	Setaria italica	1.00E-65	3.37	–
CL13350.Contig1	CHP-rich zinc finger protein-like	Oryza sativa	4.00E-35	3.72	–
Unigene18699	disease resistance protein RGA4	Aegilops tauschii	9E-15	6.91	–

Table S1 (Continued)

Gene ID	Description	Blast species	e_value	WS-9_FC	RW_FC
CL11925.Contig2	NAC domain-containing protein	Oryza sativa	1.90E-55	7.10	–
Unigene54434	NBS-LRR type protein	Oryza sativa	7.60E-10	–	7.93
**Ribosome biogenesis in eukaryotes**					
CL1258.Contig2	aldo-keto reductase	Oryza sativa	1.10E-105	2.75	–
Unigene27176	reverse transcriptase	Sorghum bicolor	4.10E-276	3.01	–
CL13686.Contig1	Retrovirus-related Pol polyprotein from transposon TNT 1-94	Nicotiana tabacum	9.60E-74	3.06	–
Unigene909	Protein kinase domain superfamily protein	Zea mays	1.00E-48	3.28	–
Unigene33764	retrotransposon protein	Oryza sativa	2.30E-54	8.10	–
CL13173.Contig3	reverse transcriptase	Oryza sativa	3.00E-22	8.49	–
Unigene65830	CCT motif family protein	Zea mays	6.00E-61	8.73	–
Unigene19471	Alliin lyase	Allium sativum	4.30E-11	8.93	–
Unigene35215	protein EMSY-LIKE 3	Sorghum bicolor	5.00E-52	9.73	–
Unigene45006	non-LTR retroelement reverse transcriptase	Oryza sativa	5.00E-23	–	8.90
**Steroid biosynthesis**					
Unigene9726	Methylsterol monooxygenase	Arabidopsis thaliana	4.40E-61	-8.92	–
Unigene28449	Achilleol B synthase	Oryza sativa	3.70E-10	2.67	–
Unigene28452	achilleol B synthase-like	Zea mays	6.02E-75	3.27	–
CL13391.Contig1	Achilleol B synthase	Oryza sativa	5.90E-35	3.33	–
Unigene518	Parkeol synthase	Oryza sativa	4.40E-11	3.60	–
Unigene28448	Cycloartenol synthase	Ricinus communis	1.50E-09	3.81	–
Unigene40051	achilleol B synthase-like	Setaria italica	6.78E-68	4.94	–
Unigene1402	cycloartenol synthase	Zea mays	1.48E-57	7.84	–
CL13693.Contig36	cycloartenol synthase	Zea mays	3.60E-226	8.12	–
Unigene28853	Cycloartenol synthase	Kalanchoe daigremontiar	8.60E-13	10.25	–
**Tyrosine metabolism**					
Unigene24406	aldehyde dehydrogenase	Saccharum officinarum	1.60E-106	2.06	–
Unigene29886	pleiotropic drug resistance protein	Zea mays	0.00E+00	2.12	–
CL629.Contig2	ABC transporter G family member	Oryza sativa	1.90E-85	3.19	–
CL11007.Contig1	ABC transporter G family member	Triticum urartu	4.50E-205	5.90	–
Unigene35203	Polyphenol oxidase	Vitis vinifera	7.50E-08	8.35	–
Unigene35089	polyphenol oxidase	Sorghum bicolor	1.70E-37	8.38	–
Unigene45639	polyphenol oxidase	Sorghum bicolor	8.20E-64	8.60	–
Unigene5329	polyphenol oxidase	Sorghum bicolor	1.00E-46	9.19	–
Unigene20571	Aromatic-L-amino-acid decarboxylase	Catharanthus roseus	8.60E-31	9.33	–
Unigene54666	ABC transporter G family member	Setaria italica	2.40E-19	–	-3.80
**Isoquinoline alkaloid biosynthesis**					
Unigene29886	pleiotropic drug resistance protein	Zea mays	0.00E+00	2.12	–
CL629.Contig2	ABC transporter G family member	Oryza sativa	1.90E-85	3.19	–
CL11007.Contig1	ABC transporter G family member	Triticum urartu	4.50E-205	5.90	–
Unigene35203	Polyphenol oxidase	Vitis vinifera	7.50E-08	8.35	–
Unigene35089	polyphenol oxidase	Sorghum bicolor	1.70E-37	8.38	–
Unigene45639	polyphenol oxidase	Sorghum bicolor	8.20E-64	8.60	–
Unigene5329	polyphenol oxidase	Sorghum bicolor	1.00E-46	9.19	–
Unigene20571	Aromatic-L-amino-acid decarboxylase	Catharanthus roseus	8.60E-31	9.33	–
Unigene54666	ABC transporter G family member	Setaria italica	2.40E-19	–	-3.80
**Cutin, suberine and wax biosynthesis**					
Unigene25289	3'-N-debenzoyl-2'-deoxytaxol N-benzoyltransferase	Taxus canadensis	3.80E-17	4.24	–
Unigene11375	3'-N-debenzoyl-2'-deoxytaxol N-benzoyltransferase	Taxus canadensis	4.00E-32	5.22	–
CL12223.Contig1	Long chain acyl-CoA synthetase	Arabidopsis thaliana	1.20E-155	5.92	–
CL12223.Contig2	Long chain acyl-CoA synthetase	Arabidopsis thaliana	8.80E-222	6.52	–
Unigene8759	2-alpha-hydroxytaxane 2-O-benzoyltransferase	Taxus cuspidata	2.70E-13	7.96	–
Unigene45671	agmatine coumaroyltransferase	Zea mays	4.50E-31	8.84	–

Table S1 (Continued)

Gene ID	Description	Blast species	e_value	WS-9_FC	RW_FC
CL12223.Contig3	Long chain acyl-CoA synthetase	Arabidopsis thaliana	1.10E-184	10.07	–
**Tryptophan metabolism**					
CL1188.Contig1	cytosolic sulfotransferase	Setaria italica	1.60E-124	-2.80	–
CL8508.Contig1	aldehyde dehydrogenase	Saccharum spontaneum	9.80E-282	3.13	–
Unigene33150	indole-2-monooxygenase	Zea mays	1.10E-14	3.19	–
Unigene18706	Cytochrome P450	Persea americana	2.40E-109	4.59	–
Unigene35446	cytosolic sulfotransferase	Setaria italica	3.50E-80	4.66	–
Unigene17341	indole-2-monooxygenase	Zea mays	1.70E-26	5.74	–
Unigene51735	Indole-3-acetaldehyde oxidase	Zea mays	1.30E-06	8.13	–
Unigene41601	Tyrosine N-monooxygenase	Sorghum bicolor	1.10E-27	–	-8.16
**Carbon fixation in photosynthetic organisms**					
Unigene26163	Fructose-bisphosphate aldolase	Oryza sativa	3.50E-120	-2.68	–
Unigene26159	Fructose-bisphosphate aldolase	Oryza sativa	4.50E-53	-2.52	–
CL6923.Contig2	Fructose-bisphosphate aldolase	Oryza sativa	3.00E-22	-2.39	–
Unigene30058	Pyruvate, phosphate dikinase 2	Oryza sativa	6.40E-17	-2.19	–
CL11737.Contig2	alanine aminotransferase	Zea mays	3.30E-249	2.21	–
Unigene21765	NADP-dependent malic enzyme	Oryza sativa	5.40E-222	7.96	–
Unigene21766	NADP-dependent malic enzyme	Phaseolus vulgaris	2.50E-58	8.30	–
CL9592.Contig2	NADP-dependent malic enzyme	Zea mays	3.70E-13	9.08	–
**Flavone and flavonol biosynthesis**					
Unigene10627	UDP-glycosyltransferase	Setaria italica	6.58E-59	-8.00	–
Unigene21145	O-methyltransferase ZRP4	Zea mays	1.20E-81	-5.05	–
CL10556.Contig1	Cytochrome P450	Arabidopsis thaliana	5.50E-160	-4.21	–
Unigene23658	Cytochrome P450	Arabidopsis thaliana	1.50E-32	-3.39	-2.60
CL7463.Contig1	Anthocyanidin 3-O-glucosyltransferase	Zea mays	1.20E-129	-3.33	–
CL13117.Contig1	O-methyltransferase-like protein	Saccharum officinarum	2.00E-198	-2.38	–
Unigene23154	indole-2-monooxygenase	Zea mays	1.40E-90	6.20	–
Unigene16462	5-pentadecatrienyl resorcinol O-methyltransferase	Sorghum bicolor	5.90E-109	–	3.07
**alpha-Linolenic acid metabolism**					
Unigene37048	4-coumarate-CoA ligase	Zea mays	3.60E-09	-9.09	–
CL1259.Contig2	Benzoate O-methyltransferase	Zea mays	4.10E-94	4.46	–
CL1259.Contig5	Benzoate O-methyltransferase	Zea mays	4.60E-44	4.53	–
CL1259.Contig1	Benzoate O-methyltransferase	Zea mays	1.60E-107	5.08	–
CL1259.Contig4	Benzoate O-methyltransferase	Zea mays	1.70E-40	5.90	–
Unigene25494	12-oxophytodienoate reductase	Oryza sativa	1.60E-103	6.17	–
**Benzoxazinoid biosynthesis**					
Unigene21145	O-methyltransferase ZRP4	Zea mays	1.20E-81	-5.05	–
CL13117.Contig1	O-methyltransferase-like protein	Saccharum officinarum	2.00E-198	-2.38	–
Unigene33150	indole-2-monooxygenase	Zea mays	1.10E-14	3.19	–
Unigene17341	indole-2-monooxygenase	Zea mays	1.70E-26	5.74	–
Unigene23154	indole-2-monooxygenase	Zea mays	1.40E-90	6.20	–
Unigene17342	indole-2-monooxygenase	Sorghum bicolor	5.00E-97	11.27	8.41
Unigene16462	5-pentadecatrienyl resorcinol O-methyltransferase	Sorghum bicolor	5.90E-109	–	3.07
**Glucosinolate biosynthesis**					
CL1188.Contig1	cytosolic sulfotransferase	Setaria italica	1.60E-124	-2.80	–
Unigene18706	Cytochrome P450	Persea americana	2.40E-109	4.59	–
Unigene35446	cytosolic sulfotransferase	Setaria italica	3.50E-80	4.66	–
Unigene41601	Tyrosine N-monooxygenase	Sorghum bicolor	1.10E-27	–	-8.16
**Glyoxylate and dicarboxylate metabolism**					
Unigene16599	D-glycerate 3-kinase	Arabidopsis thaliana	3.80E-10	3.21	–
CL5141.Contig1	glutamine synthetase	Saccharum officinarum	4.20E-23	3.41	–
Unigene19652	glutamine synthetase	Saccharum officinarum	5.43E-161	5.03	–

Table S1 (Continued)

Gene ID	Description	Blast species	e_value	WS-9_FC	RW_FC
Unigene9234	aconitate hydratase	Oryza sativa	3.10E-101	5.30	–
Unigene22101	aconitate hydratase	Ananas comosus	0.00E+00	5.66	–
Unigene19651	glutamine synthetase cytosolic isozyme	Setaria italica	1.70E-08	5.99	–
**beta-Alanine metabolism**					
Unigene25178	Beta-ureidopropionase	Triticum urartu	2.50E-216	2.08	–
CL6765.Contig1	glutamate decarboxylase	Setaria italica	1.30E-248	2.27	–
CL8508.Contig1	aldehyde dehydrogenase	Saccharum spontaneum	9.80E-282	3.13	–
**Arginine and proline metabolism**					
Unigene23719	glutamate dehydrogenase	Oryza sativa	6.50E-220	2.42	–
CL8508.Contig1	aldehyde dehydrogenase	Saccharum spontaneum	9.80E-282	3.13	–
Unigene3245	aldehyde dehydrogenase	Cleistogenes songorica	1.00E-302	3.27	–
CL5141.Contig1	glutamine synthetase	Saccharum officinarum	4.20E-23	3.41	–
Unigene19652	glutamine synthetase	Saccharum officinarum	5.43E-161	5.03	–
Unigene19651	glutamine synthetase cytosolic isozyme	Setaria italica	1.70E-08	5.99	–
**Fatty acid elongation**					
CL5162.Contig1	acyltransferase precursor	Zea mays	7.40E-212	-4.64	–
Unigene29883	3-ketoacyl-CoA synthase 6	Arabidopsis thaliana	4.70E-125	-2.63	–
Unigene51247	3-ketoacyl-CoA synthase	Arabidopsis thaliana	1.40E-10	5.32	–
**Butanoate metabolism**					
Unigene24406	aldehyde dehydrogenase	Saccharum officinarum	1.60E-106	2.06	–
CL6765.Contig1	glutamate decarboxylase	Setaria italica	1.30E-248	2.27	–
Unigene36693	11-beta-hydroxysteroid dehydrogenase-like	Arabidopsis thaliana	2.40E-38	8.50	–
**Diterpenoid biosynthesis**					
Unigene18138	Sex determination protein tasselseed	Zea mays	3.20E-46	-2.93	–
Unigene36693	11-beta-hydroxysteroid dehydrogenase-like	Arabidopsis thaliana	2.40E-38	8.50	–
Unigene12484	Ent-kaur-16-ene synthase	Oryza sativa	1.20E-217	9.56	–
**Histidine metabolism**					
CL8508.Contig1	aldehyde dehydrogenase	Saccharum spontaneum	9.80E-282	3.13	–
Unigene6248	Serine decarboxylase	Oryza sativa	6.00E-36	7.99	–
**Cyanoamino acid metabolism**					
CL10140.Contig2	beta-glucosidase	Setaria italica	0.00E+00	3.38	–
Unigene33285	cytokinin-O-glucosyltransferase	Zea mays	1.40E-33	-3.60	–
CL10639.Contig5	beta-glucosidase	Sorghum bicolor	6.00E-51	-3.05	–
Unigene27838	Beta-glucosidase 45	Arabidopsis thaliana	1.30E-23	-2.36	–
CL2285.Contig8	exhydrolase II	Zea mays	3.00E-284	4.29	–
CL7850.Contig1	Beta-glucosidase	Oryza sativa	7.70E-193	5.49	–
Unigene23154	indole-2-monooxygenase	Zea mays	1.40E-90	6.20	–
Unigene41601	Tyrosine N-monooxygenase	Sorghum bicolor	1.10E-27	–	-8.16
**Lysine degradation**					
CL8508.Contig1	aldehyde dehydrogenase	Saccharum spontaneum	9.80E-282	3.13	–
Unigene30344	Alpha-aminoadipic semialdehyde synthase	Arabidopsis thaliana	9.50E-20	3.23	–
Unigene30342	lysine-ketoglutarate reductase/saccharopine dehydrogenase	Zea mays	0.00E+00	3.55	–
**Ascorbate and aldarate metabolism**					
CL8508.Contig1	aldehyde dehydrogenase	Saccharum spontaneum	9.80E-282	3.13	–
Unigene29991	laccase-9	Oryza sativa	7.30E-50	7.15	–
Unigene29992	laccase-9	Zea mays	0.00E+00	8.69	–
**Fructose and mannose metabolism**					
CL6923.Contig2	Fructose-bisphosphate aldolase	Oryza sativa	3.00E-22	-2.39	–
Unigene26163	Fructose-bisphosphate aldolase	Oryza sativa	3.50E-120	-2.68	–
Unigene26159	Fructose-bisphosphate aldolase	Oryza sativa	4.50E-53	-2.52	–
CL7484.Contig2	plant/MUD21-2 protein	Zea mays	5.00E-171	8.18	–
CL3464.Contig2	EG45-like domain containing protein	Citrus jambhiri	5.10E-33	8.55	–

Table S1 (Continued)

Gene ID	Description	Blast species	e_value	WS-9_FC	RW_FC
Unigene15413	DUF679 domain membrane protein	Zea mays	4.00E-103	8.72	–
CL5005.Contig1	expansin-B3-like	Zea mays	2.00E-135	–	3.55
**Pentose phosphate pathway**					
CL6923.Contig2	Fructose-bisphosphate aldolase	Oryza sativa	3.00E-22	-2.39	–
Unigene26163	Fructose-bisphosphate aldolase	Oryza sativa	3.50E-120	-2.68	–
Unigene26159	Fructose-bisphosphate aldolase	Oryza sativa	4.50E-53	-2.52	–
CL7484.Contig2	plant/MUD21-2 protein	Zea mays	5.00E-171	8.18	–
CL3464.Contig2	EG45-like domain containing protein	Citrus jambhiri	5.10E-33	8.55	–
Unigene15413	DUF679 domain membrane protein	Zea mays	4.00E-103	8.72	–
CL5005.Contig1	expansin-B3-like	Zea mays	2.00E-135	–	3.55
**Glycerolipid metabolism**					
Unigene19383	Glycerol-3-phosphate 2-O-acyltransferase	Arabidopsis thaliana	3.10E-107	2.58	–
CL8508.Contig1	aldehyde dehydrogenase	Saccharum spontaneum	9.80E-282	3.13	–
Unigene19384	glycerol-3-phosphate 2-O-acyltransferase	Setaria italica	1.90E-110	3.19	–
Unigene16599	D-glycerate 3-kinase	Arabidopsis thaliana	3.80E-10	3.21	–
Unigene18840	Glycerol-3-phosphate acyltransferase	Arabidopsis thaliana	8.70E-37	4.55	–
**Monoterpenoid biosynthesis**					
Unigene6760	Short-chain dehydrogenase/reductase	Arabidopsis thaliana	2.20E-11	3.48	–
Unigene24990	Salutaridine reductase	Papaver bracteatum	7.70E-70	5.55	–
**Anthocyanin biosynthesis**					
CL7463.Contig1	Anthocyanidin 3-O-glucosyltransferase	Zea mays	1.20E-129	-3.33	–
**Taurine and hypotaurine metabolism**					
CL6765.Contig1	glutamate decarboxylase	Setaria italica	1.30E-248	2.27	–
**Galactose metabolism**					
Unigene27838	Beta-glucosidase 45	Arabidopsis thaliana	1.30E-23	-2.36	–
CL131.Contig3	soluble acid invertase	Saccharum officinarum	0.00E+00	2.12	–
CL5361.Contig8	galactinol-sucrose galactosyltransferase	Arabidopsis thaliana	1.50E-278	4.14	–
CL7484.Contig2	plant/MUD21-2 protein	Zea mays	5.00E-171	8.18	–
CL3464.Contig2	EG45-like domain containing protein	Citrus jambhiri	5.10E-33	8.55	–
Unigene15413	DUF679 domain membrane protein	Zea mays	4.00E-103	8.72	–
CL5005.Contig1	expansin-B3-like	Zea mays	2.00E-135	–	3.55
**Sulfur metabolism**					
CL1188.Contig1	cytosolic sulfotransferase	Setaria italica	1.60E-124	-2.80	–
CL4359.Contig11	cysteine synthase	Zea mays	1.00E-16	8.27	–
**Carotenoid biosynthesis**					
Unigene18138	Sex determination protein tasselseed	Zea mays	3.20E-46	-2.93	–
Unigene24010	FAD-dependent urate hydroxylase	Acinetobacter baylyi	4.00E-06	2.59	–
CL8103.Contig1	9-cis-epoxycarotenoid dioxygenase	Saccharum officinarum	0.00E+00	3.56	–
CL12417.Contig2	GSDL-motif lipase	Zea mays	0.00E+00	3.57	–
Unigene93	beta-carotene 3-hydroxylase	Zea mays	0.00E+00	4.44	–
**Pyruvate metabolism**					
Unigene30058	Pyruvate, phosphate dikinase 2	Oryza sativa	6.40E-17	-2.19	–
CL8508.Contig1	aldehyde dehydrogenase	Saccharum spontaneum	9.80E-282	3.13	–
Unigene21765	NADP-dependent malic enzyme	Oryza sativa	5.40E-222	7.96	–
Unigene21766	NADP-dependent malic enzyme	Phaseolus vulgaris	2.50E-58	8.30	–
CL9592.Contig2	NADP-dependent malic enzyme	Zea mays	3.70E-13	9.08	–
**Glycolysis/Gluconeogenesis**					
CL3464.Contig2	EG45-like domain containing protein	Citrus jambhiri	5.10E-33	8.55	–
CL6923.Contig2	Fructose-bisphosphate aldolase	Oryza sativa	3.00E-22	-2.39	–
Unigene26163	Fructose-bisphosphate aldolase	Oryza sativa	3.50E-120	-2.68	–
Unigene26159	Fructose-bisphosphate aldolase	Oryza sativa	4.50E-53	-2.52	–
CL8508.Contig1	aldehyde dehydrogenase	Saccharum spontaneum	9.80E-282	3.13	–

Table S1 (Continued)

Gene ID	Description	Blast species	e_value	WS-9_FC	RW_FC
Unigene22027	2,3-bisphosphoglycerate-independent phosphoglycerate mutase	Ricinus communis	3.90E-268	5.90	–
CL7484.Contig2	plant/MUD21-2 protein	Zea mays	5.00E-171	8.18	–
Unigene15413	DUF679 domain membrane protein	Zea mays	4.00E-103	8.72	–
CL5005.Contig1	expansin-B3-like	Zea mays	2.00E-135	–	3.55
**Natural killer cell mediated cytotoxicity**					
CL3108.Contig2	bZIP transcription factor	Setaria italica	2.20E-33	2.66	–
CL12321.Contig3	Basic leucine zipper	Arabidopsis thaliana	5.10E-10	2.74	–
**Synthesis and degradation of ketone bodies**					
Unigene36693	11-beta-hydroxysteroid dehydrogenase-like	Arabidopsis thaliana	2.40E-38	8.50	–
**Phagosome**					
CL987.Contig1	Senescence-specific cysteine protease SAG39	Oryza sativa	5.80E-91	-3.78	–
CL987.Contig2	Senescence-specific cysteine protease SAG39	Oryza sativa	6.40E-91	-3.63	–
Unigene39496	protein YLS9-like	Zea mays	2.30E-68	8.02	–
Unigene20008	KDEL-tailed cysteine endopeptidase	Zea mays	3.00E-173	10.30	–
Unigene20007	vignain	Setaria italica	1.60E-77	10.35	–
Unigene12601	cysteine proteinase	Zea mays	1.60E-17	–	8.89
**Photosynthesis-antenna proteins**					
Unigene15853	chlorophyll a-b binding protein	Zea mays	7.10E-08	-3.40	–
Unigene33096	chlorophyll a-b binding protein	Oryza brachyantha	2.05E-19	–	2.96
**Vitamin B6 metabolism**					
CL1258.Contig2	aldo-keto reductase	Oryza sativa	1.10E-105	2.75	–
**Zeatin biosynthesis**					
Unigene10627	UDP-glycosyltransferase	Setaria italica	6.58E-59	-8.00	–
Unigene28366	Zea mays putative cytochrome P450 superfamily protein	Zea mays	0.00E+00	-2.87	–
CL13686.Contig1	Retrovirus-related Pol polyprotein from transposon TNT 1-94	Nicotiana tabacum	9.60E-74	3.06	–
Unigene30973	Cytochrome P450	Arabidopsis thaliana	7.20E-78	8.35	–
**Other glycan degradation**					
Unigene30731	alpha-mannosidase	Vitis vinifera	3.70E-06	-4.18	–
CL12417.Contig2	GSDL-motif lipase	Zea mays	0.00E+00	3.57	–
**Glycine, serine and threonine metabolism**					
CL8508.Contig1	aldehyde dehydrogenase	Saccharum spontaneum	9.80E-282	3.13	–
Unigene16599	D-glycerate 3-kinase	Arabidopsis thaliana	3.80E-10	3.21	–
Unigene22027	2,3-bisphosphoglycerate-independent phosphoglycerate mutase	Ricinus communis	3.90E-268	5.90	–
**Fatty acid biosynthesis**					
Unigene36693	11-beta-hydroxysteroid dehydrogenase-like	Arabidopsis thaliana	2.40E-38	8.50	–
**Biosynthesis of unsaturated fatty acids**					
Unigene36693	11-beta-hydroxysteroid dehydrogenase-like	Arabidopsis thaliana	2.40E-38	8.50	–
**Base excision repair**					
CL11369.Contig1	dehydrin HIRD11	Sorghum bicolor	0.00E+00	2.23	–
Unigene2767	nuclear factor YB2	Sorghum bicolor	2.50E-80	2.36	–
**Lysine biosynthesis**					
CL8508.Contig1	aldehyde dehydrogenase	Saccharum spontaneum	9.80E-282	3.13	–
**Cysteine and methionine metabolism**					
CL177.Contig1	AP2-associated protein kinase	Actinidia chinensis	4.00E-16	2.28	–
Unigene23050	Zeamatin	Zea mays	3.20E-121	2.96	–
Unigene8718	1-aminocyclopropane-1-carboxylate oxidase 1	Oryza sativa	4.40E-13	8.20	–
CL4359.Contig11	cysteine synthase	Zea mays	1.00E-16	8.27	–
**Arachidonic acid metabolism**					
Unigene24567	Probable phospholipid hydroperoxide glutathione peroxidase	Spinacia oleracea	9.90E-18	-3.29	–
**Pantothenate and CoA biosynthesis**					
Unigene25178	Beta-ureidopropionase	Triticum urartu	2.50E-216	2.08	–
**Nucleotide excision repair**					

Table S1 (Continued)

Gene ID	Description	Blast species	e_value	WS-9_FC	RW_FC
Unigene2767	nuclear factor YB2	Sorghum bicolor	2.50E-80	2.36	–
Unigene19686	loricrin	Saccharum officinarum	6.90E-17	3.05	–
Unigene15869	BTB/POZ domain-containing protein	Arabidopsis thaliana	4.10E-63	6.69	–
Unigene30497	sulfate transporter 3.4	Arabidopsis thaliana	2.30E-21	9.27	–
**Citrate cycle (TCAcycle)**					
Unigene9234	aconitate hydratase	Oryza sativa	3.10E-101	5.30	–
Unigene22101	aconitate hydratase	Ananas comosus	0.00E+00	5.66	–
**SNARE interactions in vesicular transport**					
Unigene39496	protein YLS9-like	Zea mays	2.30E-68	8.02	–
**Propanoate metabolism**					
CL8508.Contig1	aldehyde dehydrogenase	Saccharum spontaneum	9.80E-282	3.13	–
**Porphyrin and chlorophyll metabolism**					
Unigene28427	chlorophyll(ide) b reductase NYC1	Oryza sativa	2.00E-254	2.34	–
CL8408.Contig1	Red chlorophyll catabolite reductase	Hordeum vulgare	6.50E-58	8.39	–
Unigene22331	Protochlorophyllide reductase A	Triticum aestivum	2.00E-35	–	2.58
Unigene22334	Protochlorophyllide reductase A	Triticum aestivum	8.60E-17	–	2.29
**Oxidative phosphorylation**					
Unigene14415	Plasma membrane ATPase	Solanum lycopersicum	1.80E-84	3.05	–
CL876.Contig2	Plasma membrane ATPase	Solanum lycopersicum	0.00E+00	3.15	–
Unigene182	ATPase 11, plasma membrane-type	Arabidopsis thaliana	1.20E-76	3.34	–
CL3464.Contig2	EG45-like domain containing protein	Citrus jambhiri	5.10E-33	8.55	–
**Glutathione metabolism**					
Unigene24567	Probable phospholipid hydroperoxide glutathione peroxidase	Spinacia oleracea	9.90E-18	-3.29	–
Unigene17126	glutathione S-transferase GSTU6	Setaria italica	1.00E-13	3.14	–
Unigene24625	glutathione S-transferase GSTU6	Zea mays	1.70E-21	3.20	–
Unigene21221	glutathione S-transferase GSTU6	Sorghum bicolor	2E-180	5.81	–
**DNA replication**					
Unigene2767	nuclear factor YB2	Sorghum bicolor	2.50E-80	2.36	–
Unigene19686	loricrin	Saccharum officinarum	6.90E-17	3.05	–
**Terpenoid backbone biosynthesis**					
CL13686.Contig1	Retrovirus-related Pol polyprotein from transposon TNT 1-94	Nicotiana tabacum	9.60E-74	3.06	–
**Ubiquitin mediated proteolysis**					
CL367.Contig1	atypical receptor-like kinase MARK	Zea mays	0.00E+00	2.96	–
Unigene21956	inactive receptor kinase RLK902	Arabidopsis thaliana	6.70E-37	4.44	–
Unigene4747	U-box domain-containing protein	Arabidopsis thaliana	1.10E-19	8.02	–
**RNA degradation**					
Unigene27176	reverse transcriptase	Sorghum bicolor	4.10E-276	3.01	–
CL13686.Contig1	Retrovirus-related Pol polyprotein from transposon TNT 1-94	Nicotiana tabacum	9.60E-74	3.06	–
Unigene909	Protein kinase domain superfamily protein	Zea mays	1.00E-48	3.28	–
Unigene24419	Amino acid permease 8	Arabidopsis thaliana	3.60E-20	5.27	–
Unigene33764	retrotransposon protein	Oryza sativa	2.30E-54	8.10	–
CL13173.Contig3	reverse transcriptase	Oryza sativa	3.00E-22	8.49	–
Unigene35215	protein EMSY-LIKE 3	Sorghum bicolor	5.00E-52	9.73	–
Unigene45006	non-LTR retroelement reverse transcriptase	Oryza sativa	5.00E-23	–	8.90
**Circadian rhythm - plant**					
CL4264.Contig4	chalcone synthase	Sorghum bicolor	1.00E-88	9.81	9.63
**Ribosome**					
Unigene44047	30S ribosomal protein S3	Oryza sativa	1.30E-06	4.99	–
Unigene50519	60S ribosomal protein L10-2	Arabidopsis thaliana	4.90E-18	7.96	–
Unigene25414	50S ribosomal protein L24, chloroplastic	Arabidopsis thaliana	3.10E-54	–	2.15
Unigene20789	50S ribosomal protein L29	Zea mays	1.80E-64	–	2.21
Unigene45434	ribosomal protein S18	Tenaxia guillarmodiae	3.40E-37	–	2.52

Table S1 (Continued)

Gene ID	Description	Blast species	e_value	WS-9_FC	RW_FC
Unigene21864	50S ribosomal protein L18	Oryza sativa	7.70E-66	–	2.55
**Phosphatidylinositol signaling system**					
CL8093.Contig1	Calmodulin-binding protein	Arabidopsis thaliana	4.60E-83	2.82	–
Unigene7945	inositol-tetrakisphosphate 1-kinase	Sorghum bicolor	8.00E-21	-8.43	–
**Mismatch repair**					
Unigene19686	loricrin	Saccharum officinarum	6.90E-17	3.05	–
**Pentose and glucuronate interconversions**					
CL11154.Contig1	Thaumatin-like protein	Pyrus pyrifolia	4.90E-64	-5.59	–
CL11154.Contig2	Thaumatin-like protein	Pyrus pyrifolia	4.90E-64	-3.82	–
Unigene26939	E3 ubiquitin ligase BIG BROTHER-related	Sorghum bicolor	8.00E-70	3.11	–
Unigene191	F-box/kelch-repeat protein	Setaria italica	0.00E+00	4.28	–
Unigene17860	vegetative cell wall protein gp1	Setaria italica	4.50E-14	7.10	–
**Fatty acid metabolism**					
CL8508.Contig1	aldehyde dehydrogenase	Saccharum spontaneum	9.80E-282	3.13	–
**Homologous recombination**					
Unigene19686	loricrin	Saccharum officinarum	6.90E-17	3.05	–
CL10938.Contig1	polyprotein	Sorghum bicolor	9.30E-171	3.77	–
**Ubiquinone and other terpenoid-quinone biosynthesis**					
Unigene37048	4-coumarate-CoA ligase	Zea mays	3.60E-09	-9.09	–
Unigene21937	2-succinylbenzoate-CoA ligase	Arabidopsis thaliana	4.70E-72	–	2.76
**RNA polymerase**					
CL11925.Contig2	NAC domain-containing protein	Oryza sativa	1.90E-55	7.10	–
Unigene25808	aspartic proteinase nepenthesin	Setaria italica	1.70E-94	2.31	–
Unigene24235	Disease resistance protein RGA2	Solanum bulbocastanum	2.10E-08	2.58	–
CL4351.Contig1	Disease resistance protein RGA2	Solanum bulbocastanum	6.30E-10	2.67	–
Unigene1583	galactinol synthase	Setaria italica	1.00E-65	3.37	–
CL13350.Contig1	CHP-rich zinc finger protein-like	Oryza sativa	4.00E-35	3.72	–
Unigene18699	disease resistance protein RGA4	Aegilops tauschii	9E-15	6.91	–
Unigene54434	NBS-LRR type protein	Oryza sativa	7.60E-10	–	7.93
**Photosynthesis**					
Unigene20163	Photosystem II 10 kDa polypeptide	Hordeum vulgare	9.70E-39	2.99	-3.32
**Valine, leucine and isoleucine degradation**					
CL8508.Contig1	aldehyde dehydrogenase	Saccharum spontaneum	9.80E-282	3.13	–
Unigene17683	methylglutaconyl-CoA hydratase	Sorghum bicolor	8.87E-93	–	-4.82
**Inositol phosphate metabolism**					
Unigene7945	inositol-tetrakisphosphate 1-kinase	Sorghum bicolor	8.00E-21	-8.43	–
Unigene38762	phosphoesterase	Saccharum officinarum	7.00E-20	9.07	–
**Brassinosteroid biosynthesis**					
Unigene30973	Cytochrome P450	Arabidopsis thaliana	7.20E-78	8.35	–
CL6280.Contig2	cycloartenol-C-24-methyltransferase	Setaria italica	2.70E-165	–	2.64

Gene ID: unigene access number of RNA-Seq; e-value: e-value from BlastX; WS-9/RW_FC indicated fold change of DEGs under water stress/rewatering.

Table S2 Validation of selected candidate genes using real time-PCR

Gene access No.	Description	Primer	Treatments	RNA-Seq	qRT-PCR
EF189713	Glyceraldehyde-3-phosphate dehydrogenase (GAPDH)	F: TGGTGCTGACTATGTCGTGGA / R: CATGGGTGCATCTTTGCTTG			
Unigene1906	Transposon protein CACTA, En/Spm sub-class	F: CACCGTGATGGAGTACGAGG / R: CCACCCACATCCCCATCATC	WS-9	3.27	3.84
			RW	3.21	2.93
CL5034.Contig1	Pathogenesis-related protein	F: ACGGCGAGAACATCTTCTGG / R: TGTTGGTGTCGTGGTCGTAG	WS-9	6.09	6.92
			RW	3.87	3.23
Unigene22524	Calcium-binding protein CML30	F: AATCATCTGATCCGGCGGTC / R: TGATCGATGCGTATGACGGG	WS-9	2.92	2.65
			RW	2.69	3.12
Unigene20102	Polcalcin Jun o 2	F: GTTCAAGCTCATGATGGCCG / R: TTTGTGGGAGCAGAGCAGAG	WS-9	3.02	4.73
			RW	2.45	3.18
Unigene58833	Disease resistance protein RPS2	F: AGCTCCCATTTGATAGCACACA / R: GTTGGGATCCCTACCCCTGA	WS-9	8.75	7.91
			RW	7.88	8.14
Unigene23660	Cytochrome P450 superfamily protein	F: GGAGCCCGTACGCGGACTCC / R: GCACCGCAAGCTCGCGGCG	WS-9	-3.40	-2.13
			RW	-3.41	-3.45
Unigene23658	Cytochrome P450	F: GAGGTCCGAGCCCATTATCG / R: ATGGTGAACATGTGGGCCAT	WS-9	-3.39	-4.16
			RW	-2.60	-2.37
Unigene20561	Flowering promoting factor-like	F: TACCTCTACGACCCCTGCC / R: ACATGTGGACGAGCTTGAGG	WS-9	-4.61	-3.16
			RW	-4.58	-3.22
Unigene23719	Glutamate dehydrogenase	F: AGGAGACGAGACGAGACGAA / R: TTAATACAGCCCGGCGGTTT	WS-9	2.42	3.47
Unigene14845	Glutamate synthase 2	F: GTTTGGGCAATTGCAGAGGG / R: AGACATTCACATGGCCCTGG	RW	2.59	2.98
Unigene21574	Heat shock protein	F: CACAGTCCACAGCTACTGCA / R: TGCTGGCATGGAAGAGGATG	WS-9	3.17	3.60
Unigene6969	Lecithin-cholesterol acyltransferase	F: AGTTCCTCTGCTTCGTGACG / R: CAGATGATCTACGTCCCGGC	RW	-7.88	-8.04
Unigene23962	Protein FAF-like	F: CTCTCCTTCGTCGAGTGCTC / R: CCCTGTCCACCACCTCAATC	WS-9	-3.19	-3.63
Unigene28610	Glucan endo-1,3-beta-glucosidase 1-like	F: TTGCAGAGAAGGAAGGCGAG / R: AGGAGTCGCTAGCACAACAC	WS-9	4.45	4.17
Unigene27617	Expansin	F: GCATTGTTGTTCACGGTCCC / R: AGGGGAGTGGTGTCGAGTAA	RW	5.92	5.24

Note: Primers of F or R represent forward or reverse primers, treatments WS-9 and RW represent water stress and re-watering, number in RNA-Seq or qRT-PCR represented fold change of differential expressed genes in two testing methods, respectively.
